# Harnessing Nonlinear Mechanics to Transform Medical Diagnostics

**DOI:** 10.1115/1.4070319

**Published:** 2025-11-01

**Authors:** C. Nataraj, Sadra Hemmati

**Affiliations:** Villanova Center for Intelligent Learning Systems, Villanova University, Villanova, PA 19085; Department of Mechanical Engineering, Villanova University, Villanova, PA 19085; Villanova University; Villanova University

**Keywords:** nonlinear dynamics, nonlinear mechanics, precision medicine, medical diagnostics, review

## Abstract

Medical diagnostics continues to be one of the most difficult challenges in healthcare, with diagnostic errors constituting the most common, costly, and harmful category of medical errors. They contribute to millions of adverse outcomes globally each year. The principal difficulty lies in the extraordinary complexity of the human body, a multiscale, adaptive, nonlinear dynamical system whose hidden states defy simplifications and contradict intuitive thinking. Current practice, largely dependent on heuristic guidelines, physician judgment, and black box machine learning, remains fundamentally limited, perpetuating diagnostic failures and preventing true personalization. This paper argues that nonlinear mechanics and dynamics are not just refinements but essential to understanding physiology. Nonlinear phenomena such as instabilities, bifurcations, chaos, fractals, adaptive feedback, and multiscale interactions occur across all the systems in the body including cardiovascular, respiratory, metabolic, neural, immune, and musculoskeletal subsystems, and are central to both health and disease. Ignoring these phenomena costs us mechanistic understanding and puts accurate diagnostics out of reach. At the same time, mechanistic models, data-driven Artificial Intelligence, and physician expertise each have unique strengths but are inadequate when applied in isolation. We propose their synthesis through physics-informed machine learning, hybrid frameworks, and the emerging paradigm of digital twins. Such systems combine mechanistic insights, data-driven computations, and experiential clinical wisdom to deliver interpretable and personalized diagnostics. Importantly, embedding nonlinear mechanics in real-time, patient-specific, hybrid models provides an exciting path toward reducing errors, improving outcomes, and transitioning from reactive, guideline-driven practice to truly pro-active, precision medicine.

## 1 Introduction and Background

Mathematical modeling has always been the backbone of engineering disciplines, firmly grounded in physics, chemistry, and allied sciences. The extraordinary advances of engineering in the twentieth century, ranging from aerospace and robotics to computing and biomedical devices, demonstrate the power of modeling as a driver of innovation and impactful societal transformation. Over the past five decades, nonlinear solid mechanics, dynamics, and fluid mechanics (hereafter “nonlinear mechanics”) have taken center stage in analysis, design, diagnostics, and control. Nonlinear methods not only improve quantitative accuracy but also explain behaviors that cannot even be qualitatively captured by linearized theories, such as instabilities, bifurcations, limit cycles, chaos, and multiperiodic responses. Despite these successes, nonlinear approaches are still not as widely adopted in engineering practice as they could be, and efforts continue to encourage their broader integration.

In medicine, by contrast, the role of mathematical modeling has historically been far more limited. Although medicine is enriched by scientific knowledge and empirical findings, the systematic application of modeling, in particular, nonlinear modeling, has not had a transformative effect comparable to engineering. This is unfortunate, as accurate modeling could fulfill several critical roles:

- Uncovering mechanisms that govern physiological and pathological processes.
- Explaining puzzling clinical observations and guiding hypothesis generation.
- Validating or discriminating among competing mechanistic explanations.
- Providing a platform for *in silico* experimentation, reducing the cost, time, logistical complexity, and ethical concerns of *in vivo* and *in vitro* studies.

Yet, despite these opportunities, biomedical modeling lags far behind engineering applications, largely due to one overwhelming obstacle: *complexity*.

Among all natural and engineered systems, the human body is arguably the most complex dynamical system. An average human body of 70 kilograms contains approximately $3\times 10^{13}$ cells (or $10^{28}$ atoms), integrated into a vast network of nonlinear subsystems operating across multiple spatial levels from cells to tissues, organs, and systems, and temporal scales ranging from microseconds to decades. These subsystems interact through dense webs of feedback and adaptive control in addition to going through self-adaptations. The hallmarks of complex systems, such as emergence, sensitivity to initial conditions, unpredictability, circular causality, and strange attractors are all present in human physiology [1,2]. The body is therefore a quintessential “system of systems,” whose complexity is both daunting and fascinating.

This complexity is amplified by gaps in our fundamental understanding. Many biological mechanisms remain poorly characterized, and new evidence continually highlights unexpected cross-domain links. For example, emotional and cognitive states can directly influence immune and biochemical processes [3], while the ongoing debates around the mind–body problem and the nature of consciousness continue to challenge traditional scientific boundaries [4,5]. At first glance, such opacity may seem discouraging. Still, history does provide perspective: before the advent of mathematics and empirical laws of physics and chemistry, the physical world also appeared impossibly complex. Centuries of systematic inquiry gradually peeled away these mysteries, leading to technologies as advanced as quantum computers and interplanetary spacecraft. Similarly, sustained exploration using nonlinear modeling and carefully designed experimentation can progressively illuminate human physiology. We contend that nonlinear mechanics must be central to this effort.

### 1.1 Medical Diagnostics: A Pattern Matching Problem in a Nonlinear Domain.

Medicine is often described as a science, since it draws on vast scientific knowledge and benefits from advances in sensing, instrumentation, and pharmaceuticals. Yet, as Montgomery has argued in her provocative work [6], medicine is indeed *scientific* but, rather than being a strict science, it is better described as a form of *phronesis*, a concept rooted in Aristotelian ethics [7]. Phronesis refers to a practical wisdom rooted in judgment under uncertainty. In this sense, medical diagnostics is not simply a matter of applying fixed rules, but an art of pattern recognition that requires judgment, experience, and flexibility. Today, it is physicians, while working under constraints of time and incomplete data, are the ones who routinely perform the extraordinary task of identifying disease causes and selecting appropriate interventions.

Nevertheless, diagnostics are fraught with challenges. It involves interpreting symptoms, forming hypotheses, and mapping them onto causal mechanisms, an inherently nonlinear problem exacerbated by profound patient variability. Clinical judgment has historically been, and remains, the dominant diagnostic method [8]. Yet, public (and even professional) expectations of diagnostic infallibility often collide with reality. Diagnostic errors are now recognized as the most frequent, costly, and dangerous of all medical mistakes [9,10]. They are the third leading cause of death in the United States, contributing to nearly 10% of hospital fatalities and affecting more than 12 million adults annually [11,12]. The financial toll is equally staggering: diagnostic errors cost the U.S. economy an estimated $50–100 billion each year [8]. Errors arise not only from missed or delayed diagnoses but also from over-diagnosis, leading to unnecessary interventions such as 22% of pacemakers, 38% of knee replacements, 30% of hysterectomies, 60% of spinal surgeries, and 50% of Cesarean sections, each carrying risks of complications, long-term morbidity, and additional costs [13].

Further complicating the picture is the difficulty of defining what is “normal” versus “abnormal.” Physiologic variability, the body's adaptability to disease, and risks associated with invasive treatments blur these boundaries. Physicians must make decisions based on limited, heterogeneous information: demographics, lab tests, imaging, and dynamic signals such as heart rate, Electrocardiogram (EKG), and arterial pressure, in which critical nonlinear dynamics are often hidden from view. Cognitive biases further increase the risk of error. Availability bias, confirmation bias, overconfidence, and rule-based shortcuts can skew decisions [14–17]. Physicians are additionally constrained by the lack of consistent feedback on diagnostic accuracy, though in some circumstances, experiential learning may partially compensate.

The difficulty of diagnostics ultimately stems from the fact that the human body is a nonlinear, adaptive, dynamic system with many hidden states. Nonlinear effects are rarely intuitive [18], and as Singer has argued [19], human cognition itself may be ill-suited to grasp such complexity unaided. This limitation, combined with the body's immense variability, helps explain the persistence of diagnostic errors worldwide. The scale of the problem underscores the urgent need for systematic incorporation of nonlinear dynamics, modeling, and scientific analysis into diagnostics. By reframing diagnosis as a nonlinear systems problem, we can begin to envision new paradigms that improve accuracy, personalize care, and reduce harm.

### 1.2 Objectives.

Accurate diagnosis is extremely difficult, if not impossible, without a mechanistic understanding of disease onset and progression. It is therefore reasonable to posit that nonlinear mechanics and dynamics hold the key to significant improvements in medical diagnostics. The objective of this paper is to lay out a case for the use of mathematical modeling in medicine, with a particular emphasis on diagnostics. While the knowledge base on human physiology is incomplete, it is already sufficient to justify a systematic effort to integrate modeling into clinical workflows. Here, we provide a focused review of nonlinear mechanics in major physiological subsystems of the body, with emphasis on their diagnostic relevance. We also contrast traditional diagnostic approaches with emerging machine learning (ML) and artificial intelligence (AI) methods. Finally, we speculate on how advances in sensing, large-scale data acquisition, high-performance computing, hybrid AI and AI-mechanistic frameworks can be integrated to create digital diagnostic tools that are more accurate, robust, and personalized. Such systems hold the promise of reducing errors, improving outcomes, and ultimately saving lives.

## 2 Review of Nonlinear Mechanics in the Human Body

### 2.1 Overview.

The human body is a paradigmatic example of a nonlinear system composed of interacting subsystems such as cardiovascular, respiratory, metabolic, neural, immune, and musculoskeletal, each governed by intrinsic dynamics, and is moreover coupled through dense networks of feedback and regulation. These hierarchically organized layers, from molecules to organs to whole-organism behavior, produce emergent properties that cannot be inferred from isolated components, which is indeed a hallmark of biological complexity [20,21]. This hierarchical coupling yields both robustness and fragility: nonlinear feedback promotes adaptability under normal conditions but can amplify small perturbations into large-scale dysfunction in disease.

The application of nonlinear dynamics to physiology has a rich lineage. Winfree's studies of biological oscillators showed how feedback loops generate rhythms across organ systems [22]; Mackey and Glass introduced the concept of “dynamical diseases” arising from feedback delays [23,24]; these insights were extended to clinical contexts, identifying nonlinear variability and fractal dynamics as fundamental to health and disease [25,26]. This later led to the identification of synchronization as a unifying principle for rhythmic processes in physiology [26]. Together, these contributions initiated nonlinear mechanics as a framework linking mechanistic insight to medical diagnostics.

### 2.2 Core Nonlinear Phenomena in Physiology.

Understanding diagnostics through nonlinear dynamics requires familiarity with universal system behaviors such as instabilities, bifurcations, chaos, fractals, adaptive feedback, and multiscale coupling, which provide the conceptual language for interpreting physiological variability and disease transitions [27,28].

*Instabilities*: An instability occurs when a small perturbation in a system's state or parameters grows rather than decays, driving the system away from equilibrium. In physiology, instabilities underlie transitions such as the onset of cardiac arrhythmia, epileptic seizure, or respiratory collapse, where normal regulatory equilibria lose their capacity to restore homeostasis.

*Bifurcations*: A bifurcation denotes a qualitative change in the system's behavior as a control parameter passes a critical threshold. Small, continuous parameter variations can produce abrupt transitions between qualitatively distinct states, such as rhythmic to arrhythmic cardiac activity, illustrating how disease often reflects a shift to a new attractor regime.

*Chaos*: Chaotic systems exhibit deterministic but aperiodic behavior, extreme sensitivity to initial conditions, and a broad spectrum of temporal scales. Physiological chaos, as seen in heartbeat or neural firing variability, reflects the system's adaptability and dynamic richness; its suppression is frequently associated with disease or aging. Chaos, far from being pathological as one might readily assume, is often a hallmark of healthy adaptability: cardiac and neural systems, for instance, exploit chaotic regimes to remain flexible and resilient. In disease, this balance is lost, producing either excessive periodicity (rigidity) or disorganized randomness. Such distinctions are diagnostically valuable, as chaos analysis of physiological signals can uncover hidden transitions and bifurcation points [29].

*Fractals*: Fractals provide another lens into nonlinear physiology by describing self-similar structures across scales, where fluctuations at one timescale resemble those at others. Healthy physiological signals such as heart rate, gait, or neural activity often display fractal or multifractal scaling, reflecting hierarchical organization and efficient information propagation. Disease states frequently exhibit a breakdown of this structure, leading to either oversimplified regularity or uncorrelated noise, an observation central to the “loss of complexity” hypothesis [30]. Fractal analysis thus has the potential to be an important diagnostic tool across cardiology, neurology, sleep medicine and others.

*Feedback*: Biological systems maintain function through nested feedback loops that regulate variables over multiple time and spatial scales. Nonlinear feedback allows amplification, damping or oscillation depending on the state of the system, producing rich dynamics that enable resilience but also predispose to instability when regulation fails.

*Multiscale phenomena*: Physiological behavior emerges from interactions across molecular, cellular, organ-level, and systemic processes. These multiscale couplings permit coordination and adaptability but also propagate dysfunction: molecular perturbations can cascade upward to system-wide instability, while systemic feedback can modulate molecular expression. Capturing these cross-scale dependencies is central to a unified nonlinear dynamics framework for diagnostics.

### 2.3 Examples From Physiology.

Biological rhythms exemplify nonlinear organization. Oscillators pervade human physiology: cardiac pacemaker cells drive the heartbeat, respiratory neurons generate breathing, metabolic cycles regulate insulin, and neural oscillations coordinate and control sleep. Clinically measurable methods such as EKG, electroencephalography (EEG), electromyography (EMG), and photoplethysmography capture these rhythmic dynamics [31–34]. Perturbations to these oscillators produce “dynamical diseases” [24,35] such as arrhythmia, epilepsy, Parkinsonian tremor, etc.

**Fig. 1:**
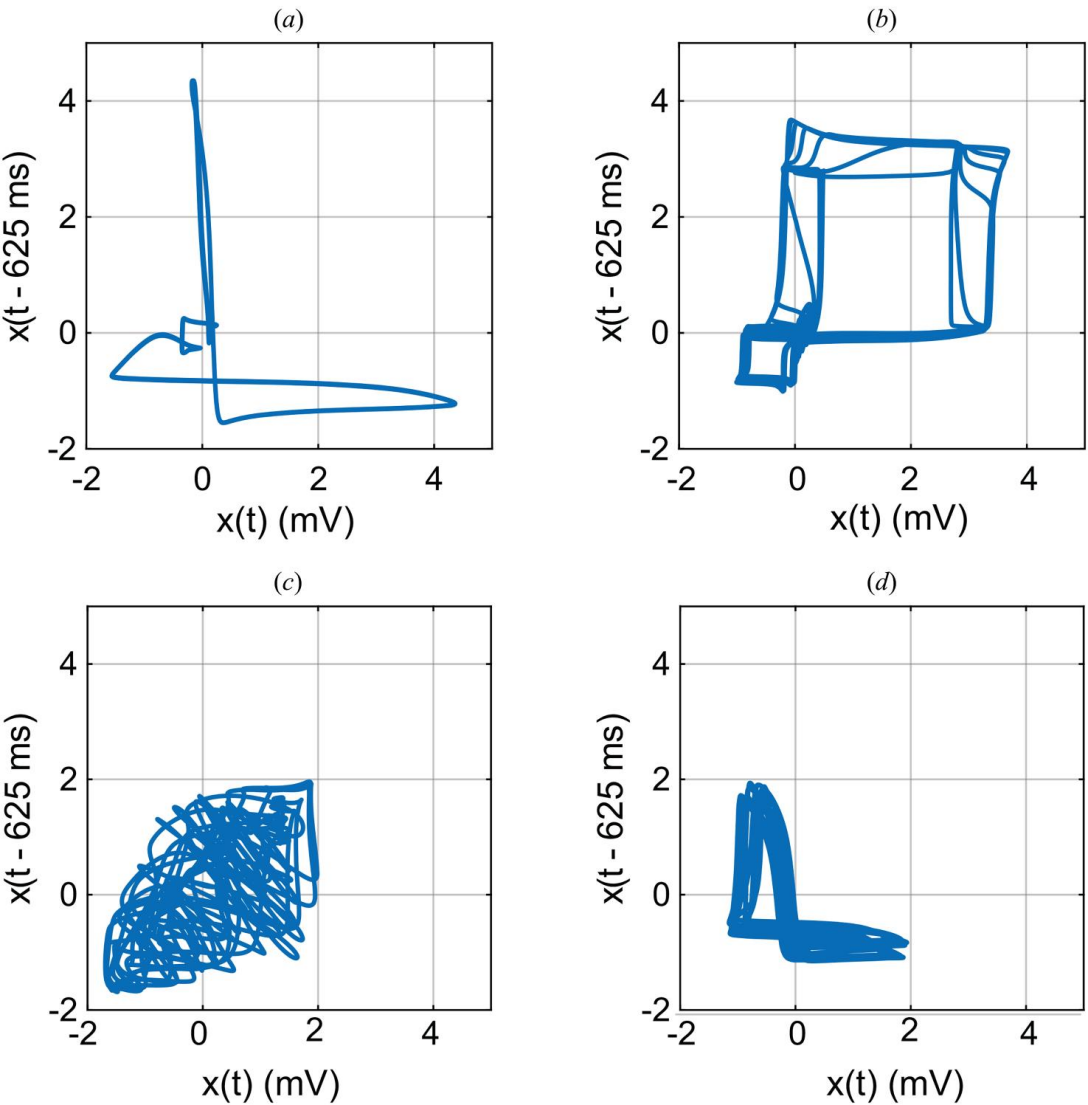
Delay embedded phase plane plots (delay = 0.625 s) in EKG: (*a*) normal, (*b*) atrial flutter, (*c*) ventricular flutter, and (*d*) atrial fibrillation

An example is the rich dynamics observed in the EKG signal. Figure 1 displays a simulated EKG signal that has been processed by a powerful nonlinear visualization method called *delay embedding*. Here, a signal is plotted against a time-delayed version of itself to reveal phase-space topology. As shown in the figure, healthy and pathological biosignals exhibit qualitatively distinct attractor geometries ranging from periodic to chaotic [36,37]. These differences could be exploited to form the basis for nonlinear diagnostic discrimination.

**Fig. 2:**
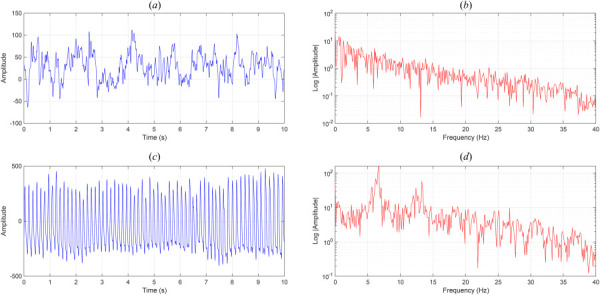
EEG waveforms (time and FFT) for (*a*–*b*) healthy individual; (*c*–*d*) epileptic individual [41]

As a second example, consider that healthy electro encephalography (EEG) activity exhibits complex, irregular, and often chaotic dynamics, reflecting the brain's adaptability and high-dimensional network behavior [38,39]. In contrast, epileptic seizures are characterized by increased synchronization and reduced dynamical complexity, where EEG signals become quasi-periodic and low-dimensional as neuronal populations enter hypersynchronous states [40,41]. This transition from chaotic richness to periodic regularity exemplifies the loss of complexity that marks many pathological state changes in physiology [42]. Figure 2 displays an example of this phenomenon, where the top row shows the EEG time response and the Fast Fourier spectrum for a healthy individual, while the bottom row shows the same for an epileptic patient. The healthy individual's waveform has a broadband structure, while the epileptic individual shows strong dominant frequencies, for example, around 6 Hz (and its second harmonic).

### 2.4 Disease as a Dynamical Transition.

Within the framework of nonlinear dynamics, a *disease* can be rigorously conceptualized not merely as the presence of abnormal measurements or biomarkers, but as a qualitative transformation in the governing dynamics of the physiological system. In this view, the healthy organism occupies a region of the system's state space characterized by stable attractors that support adaptive variability and homeodynamic regulation. Disease emerges when the underlying system experiences a *loss of stability*, a *bifurcation*, or a *transition to a new attractor* that disrupts normal regulatory behavior [43–45].

Such transitions may take several forms. A continuous (supercritical) bifurcation can gradually erode resilience, leading to subclinical dysfunctions that precede overt disease, whereas a discontinuous (subcritical) bifurcation can trigger abrupt shifts, which are analogous to the onset of arrhythmia, epileptic seizure, or respiratory failure. In other cases, excessive feedback gain or delay may induce sustained oscillations or chaotic behavior where equilibrium once prevailed. From this perspective, pathology represents not simply deviation from a normative value but a reorganization of the system's attractor landscape, wherein formerly stable equilibria become unstable, and new, maladaptive states dominate.

This definition reconciles medical and physical reasoning by identifying disease as a transition in the topology of the dynamical system's phase space. It emphasizes that diagnostics should focus on detecting early signs of instability, changing attractor geometry, or altered system sensitivity: quantities that nonlinear methods are uniquely equipped to measure. Such a formulation provides a mechanistic and mathematically grounded understanding of disease progression, unifying diverse pathologies under the general principles of dynamical transitions and stability loss. Early warning signatures such as critical slowing down, increased autocorrelation, or altered variance precede these transitions in many physiological systems.

### 2.5 Disease as Loss of Physiological Complexity.

Healthy systems display richly structured, fractal-like variability reflecting their adaptive capacity across scales. These complex temporal correlations reflect an intricate balance between order and variability and embody the system's intrinsic *adaptive capability* or its ability to respond flexibly to internal and external perturbations across multiple temporal and spatial scales. Pathological or aging systems exhibit loss of complexity, characterized by either excessive regularity (rigidity, overly periodic behavior) or randomness (uncorrelated noise) [44–46]. The “edge of chaos” metaphor captures this balance: health resides in a regime of maximal adaptability poised between these two states [47]. This has been formalized as the loss-of-complexity hypothesis: senescence and disease correspond to the breakdown of multiscale coupling among regulatory subsystems. Comparable transitions have been identified in other physiological domains. For example, in motor control and postural regulation, healthy variability follows fractal scaling laws, while pathology and aging are associated with reduced complexity and adaptability [48]. More recent studies using detrended fluctuation analysis and entropy-based metrics quantitatively confirm that loss of scaling correlations accompanies pathological conditions [49]. Thus, variability itself becomes diagnostically meaningful, and the degree and structure of fluctuations provide quantifiable indicators of system health.

### 2.6 Analytical and Computational Tools for Nonlinear Diagnostics.

Advances in nonlinear dynamics have produced a rich suite of analytical and computational tools capable of characterizing the complex variability inherent in physiological systems. These techniques extend beyond classical linear analyses by revealing the geometry of attractors, the structure of variability, and the temporal organization of fluctuations that distinguish health from disease.

A foundational approach is the reconstruction of phase space from scalar measurements through time-delay embedding, as formalized by Takens' theorem [50,51]. This allows estimation of attractor topology and computation of invariants such as Lyapunov exponents, correlation dimension, and entropy measures. The largest Lyapunov exponent quantifies sensitivity to initial conditions which is an indicator of deterministic chaos [52,53], while entropy measures capture irregularity and predictability [44,47]. Healthy systems typically exhibit intermediate entropy and positive Lyapunov exponents, reflecting structured adaptability, whereas pathological states drift toward either rigidity or randomness.

Complementary methods such as detrended fluctuation analysis (DFA) and multifractal DFA (MFDFA) quantify long-range correlations and scaling behavior in noisy biomedical signals. Healthy dynamics, such as those governing heart rate, gait, or neural activity, often follow power-law scaling with fractal exponents near unity, indicating a balance between order and disorder. Disease and aging disrupt this structure, producing flattening or narrowing of the multifractal spectrum [49,54].

Fractional calculus has recently emerged as a powerful mathematical framework for modeling physiology. By capturing memory and hereditary effects through noninteger derivatives, fractional models naturally account for viscoelastic tissue behavior, anomalous diffusion in cells, and long-range correlations in physiological signals. Applications include fractional-order models of lung mechanics, cardiovascular impedance, and neural membrane dynamics, each showing improved accuracy compared with traditional integer-order formulations [55,56]. Such approaches are especially promising for diagnostics, as they allow compact yet realistic representation of complex biological responses.

Together, these tools quantify the geometry of variability and enable diagnostics grounded in dynamics rather than static averages, bridging mechanistic models with clinical data across organ systems. Nonlinear mechanics, hence, provides a unifying framework for interpreting physiological complexity, disease onset, and adaptation. By integrating bifurcation analysis, chaos theory, fractal geometry, and data-driven computation, it reframes health and disease as transitions within dynamic landscapes, offering a rigorous, mechanistic foundation for next-generation medical diagnostics.

In the sections that follow, we examine how nonlinear approaches have advanced the specific study of cardiovascular, neuromuscular, metabolic, respiratory, immune, and neural systems, emphasizing their potential to transform medical diagnostics.

### 2.7 Cardiovascular Systems.

**Fig. 3:**
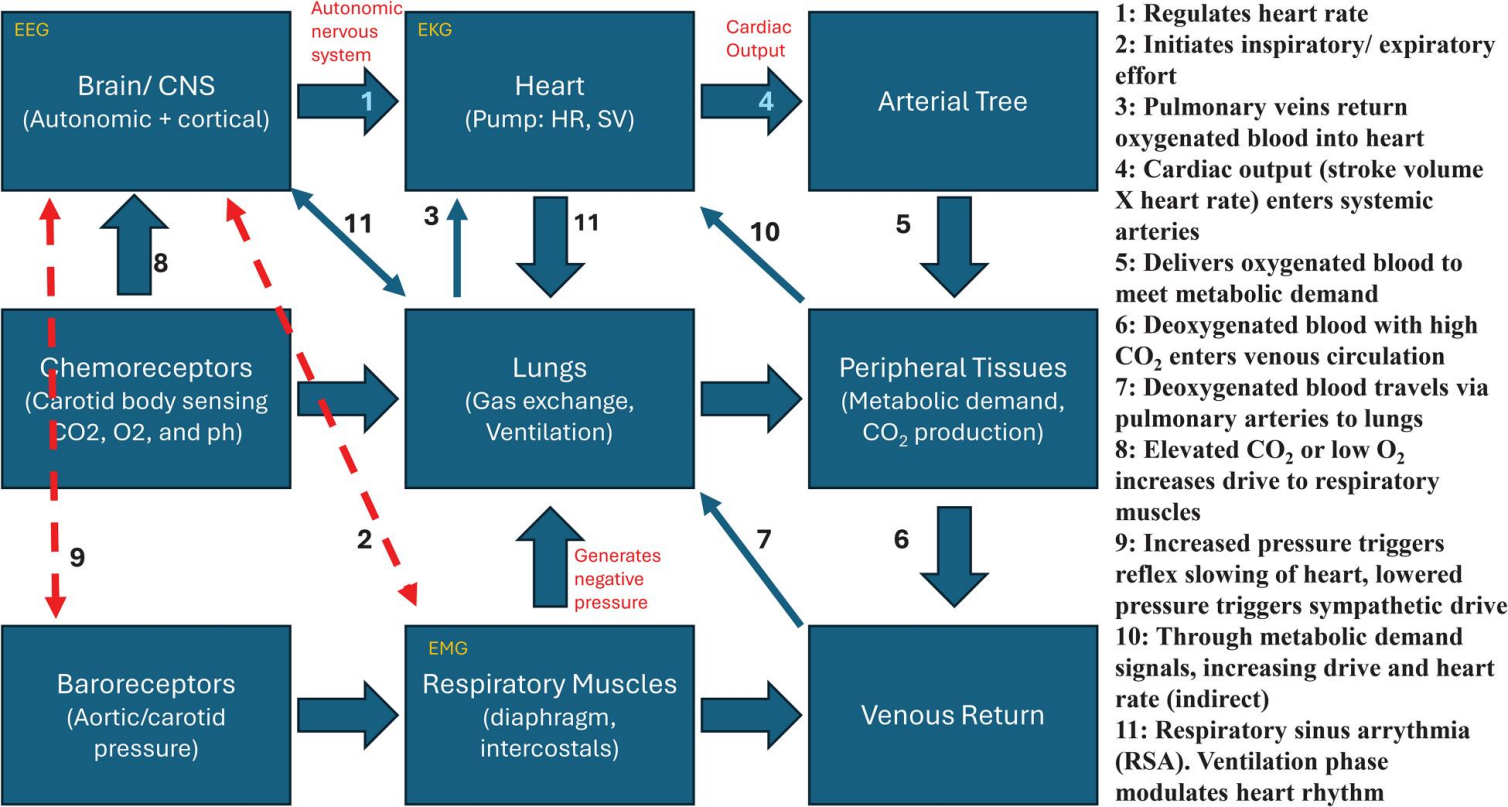
High-level overview of the cardiovascular system

The cardiovascular system has long served as a naturally attractive laboratory for nonlinear modeling, offering a unique opportunity to bridge fundamental dynamics with clinical diagnostics and therapy. As illustrated in Fig. 3, the heart and vasculature comprise a tightly coupled feedback system spanning multiple scales: ion channels and intracellular processes, excitable tissues and conduction networks, organ-level pumping, and circulation through a branching, compliant vascular system. Each level introduces nonlinearities that interact across scales, making the system particularly rich for both mechanistic modeling and diagnostic applications.

#### 2.7.1 Cellular and Tissue Models.

At the cellular level, the cardiac action potential arises from nonlinear interactions between multiple ionic currents. Early detailed ionic models, such as those of Hodgkin–Huxley type, revealed how threshold phenomena and restitution properties could generate oscillations, alternans, and chaotic behavior [57]. Simplified excitable systems such as FitzHugh–Nagumo extensions have been widely employed to explore tissue-level propagation and reentrant waves. More recent studies emphasize the role of intracellular calcium cycling, voltage–calcium feedback loops, and localized nonlinear excitations, showing how microscopic instabilities can escalate into macroscopic arrhythmias [58,59]. These models are essential for understanding mechanisms such as fibrillation, where chaotic wave breakup reflects the interplay of excitability, conduction heterogeneity, and nonlinear feedback.

#### 2.7.2 Heart Rhythm Dynamics.

Beyond detailed ionic models, the heart has been a testbed for nonlinear dynamics more broadly. Pioneering studies demonstrated that arrhythmias can be interpreted in terms of bifurcations, chaos, and low-dimensional maps that capture alternans and rhythm transitions [60,61]. Fractal variability of interbeat intervals and scaling laws in heart rate dynamics further highlight the nonlinear nature of normal physiology, with loss of complexity often linked to aging and disease [30]. These insights support diagnostic approaches based on nonlinear time-series analysis of EKG, where measures such as entropy, detrended fluctuation analysis, and recurrence quantification provide markers of autonomic function and cardiovascular risk.

#### 2.7.3 Systemic and Pulmonary Blood Flow.

The circulation is a feedback-driven system in which the heart pumps a non-Newtonian fluid through a branching, compliant vascular network (Fig. 3). Pulsatility, turbulence, wave reflections, wall compliance (including neural modulation), interindividual variability, and aging effects all complicate modeling [62–64]. Nevertheless, understanding the nonlinear mechanics of flow is crucial, since they are intimately tied to vascular health and disease.

Modeling efforts are often divided by spatial scale. Lumped-parameter models remain widely used to represent systemic and pulmonary circulation, capturing global hemodynamics via electrical analogs of resistance and capacitance [65,66]. At smaller scales, viscous forces dominate over inertia, making flow highly sensitive to vessel geometry. In this regime, the Fåhræus–Lindqvist effect reduces apparent viscosity by driving red cell migration toward the vessel axis, thereby improving perfusion [67–72].

The interaction of blood flow with vascular walls generates shear stresses that directly influence endothelial biology. Endothelial cells sense wall shear stress and respond by releasing nitric oxide and other mediators that regulate vessel tone, permeability, and remodeling [73–76]. Thus, mechanical forces arising from nonlinear flow are directly transduced into biochemical signaling pathways.

Pathophysiological states can also be interpreted through this physics framework. Stenoses, aneurysms, and valve defects alter local hemodynamics, introducing turbulence, recirculation zones, and abnormal shear stresses that accelerate vascular injury and drive disease progression [77]. Such phenomena are difficult to capture with low-order models, motivating the use of computational fluid dynamics, finite element, and finite difference methods. Patient-specific simulations now enable prediction of coronary flow reserve, shear stress distributions, and rupture risk, providing a foundation for digital twin approaches that integrate nonlinear flow mechanics with diagnostic and therapeutic planning [78–81].

#### 2.7.4 Nonlinear Signals and Diagnostics.

In parallel with mechanistic modeling, nonlinear time-series analysis of cardiac signals has become a diagnostic tool in its own right. EKG analysis using entropy, fractal measures, and symbolic dynamics has been applied to arrhythmia classification, ischemia detection, and risk stratification. Photoplethysmography signals, which capture blood volume changes in peripheral vessels, also exhibit nonlinear variability, with applications in stress monitoring, atrial fibrillation detection, and sleep studies. These diagnostic approaches complement mechanistic models, highlighting how the intrinsic nonlinear dynamics manifest directly in measurable clinical signals.

In summary, the cardiovascular system exemplifies the integration of nonlinear dynamics across scales: excitable cellular media producing complex rhythms, systemic hemodynamics shaped by wave interactions and non-Newtonian effects, and disease processes amplified by flow–structure feedback. Both mechanistic and data-driven nonlinear models are central to advancing cardiovascular diagnostics and developing precision approaches.

#### 2.7.5 Electrocardiogram-Based Modeling.

The electrocardiogram (EKG) records the electrical activity of the heart and remains central to cardiovascular diagnostics. Because many disorders manifest in the EKG, there has been sustained interest in extracting mechanistic and diagnostic insights from its dynamics. While the physiological links are not always explicit, nonlinear models and complexity-based analyses have consistently provided deeper explanatory and predictive power than linear methods.

A major foundation is the study of heart rate variability (HRV), which is a statistical measure of the interbeat interval times constructed as a time series. A landmark task force report established standards for HRV measurement [82], and subsequent nonlinear studies revealed fractal scaling and long-range correlations in heartbeat intervals that differentiate healthy from diseased states [30,83,84]. Multiscale entropy analysis [85] further showed that the loss of complexity is a robust marker of pathology. These works have become canonical in the field, and modern reviews summarize a wide variety of nonlinear measures, including recurrence plots, Lyapunov exponents, correlation dimensions, entropy metrics, and symbolic dynamics [86–89].

On the modeling side, nonlinear oscillators have been widely used to capture the essential features of cardiac dynamics. The van der Pol oscillator (capable of exhibiting self-excited limit cycle oscillations) and its extensions remain a popular choice [90,91], while fractional-order and delay models provide additional flexibility [92]. Discrete reaction–diffusion systems have been proposed to mimic conduction pathways and pacemaker behavior [93]. Nonlinear quantifiers such as Lyapunov spectra, Poincaré maps, and recurrence-based methods have proven valuable for detecting arrhythmias and characterizing bifurcations in pathological rhythms [94–96].

Clinical applications of these approaches are wide-ranging. Nonlinear HRV features have been used to stratify risk in pulmonary hypertension [97], heart failure and renal disease [98,99], and to characterize aging and healthy variability [100–102]. Complexity-based measures have aided in arrhythmia prediction [103], sudden cardiac death risk [104], and outcomes of interventions such as defibrillation or bypass surgery [105,106]. More recent work integrates nonlinear features with machine learning for automated classification and prognosis, further underscoring the translational potential of nonlinear dynamics in medical diagnostics.

Overall, nonlinear modeling and analysis of EKG signals provide a unifying framework that bridges physiology, mathematics, and clinical practice. From canonical discoveries of fractal scaling to modern hybrid AI approaches, this body of work demonstrates that complexity metrics and nonlinear models capture essential features of cardiac dynamics that remain inaccessible to traditional linear analysis.

### 2.8 Neuromuscular Systems.

**Fig. 4:**
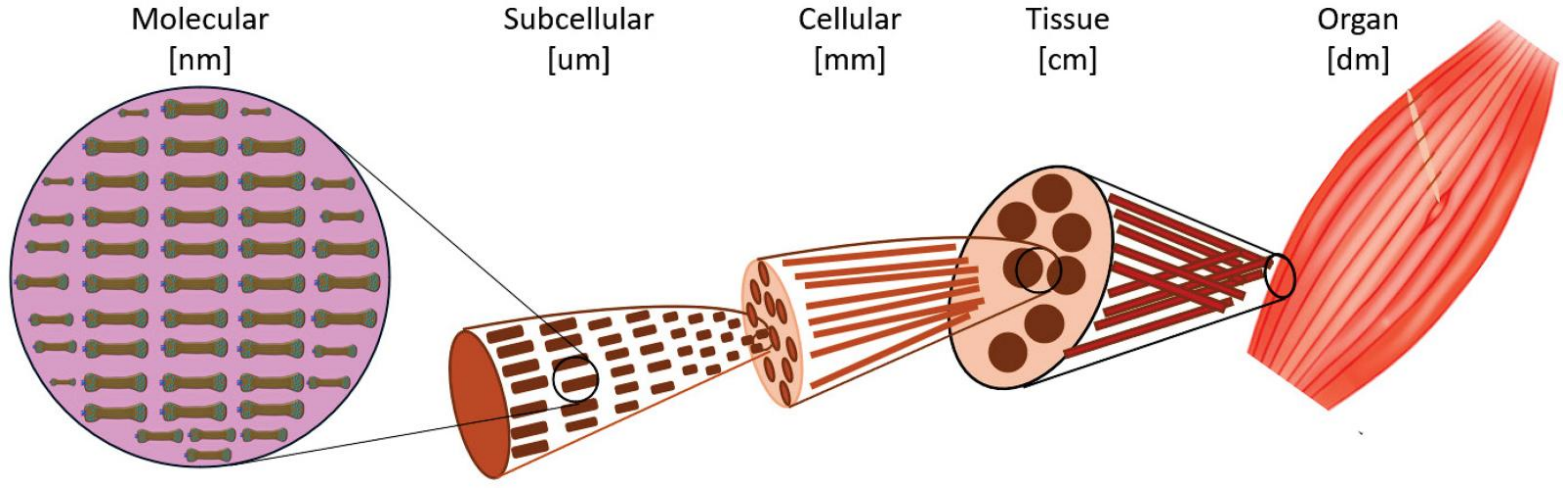
Length scales of skeletal muscle adaptation. Adapted from Ref. [107]. Used with permission.

The neuromuscular system underlies all voluntary and involuntary movements and is inherently nonlinear. This nonlinearity arises not only from the mechanics of muscles, tendons, and ligaments, but also from their coupling with the nervous system and the many sensing modalities that act as feedback control. Skeletal muscle itself is a multiscale structure, with organization spanning from the molecular to the organ level, spanning across nearly eight orders of magnitude (Fig. 4), exhibiting self-similarity that motivates the application of complexity-based analyses [107]. At the nanometer scale, contractile proteins such as actin and myosin interact within sarcomeres to generate force. These sarcomeres are arranged in series and parallel to form myofibrils, which themselves are bundled into muscle fibers surrounded by endomysium. Groups of fibers are organized into fascicles, encapsulated by perimysium, and fascicles collectively form the muscle belly, wrapped in epimysium. This hierarchical arrangement, reinforced by the extracellular matrix and tendon insertions, ensures efficient force transmission across scales. Adaptation to mechanical stimuli thus reflects the coordinated response of molecular, cellular, and tissue-level structures, linking nanometer-scale protein interactions to whole-organ function [108].

At the macroscopic scale, it has long been recognized that muscles, tendons, and ligaments behave as materials with nonlinear elasticity and damping [109]. More recent work confirms that muscles exhibit viscoelasticity governed by nonlinear constitutive laws [110]. Large-scale computational models of musculoskeletal systems have also been developed, often motivated by robotics [111], but these approaches could equally be leveraged for human diagnostics and personalized biomechanics. This framework sets the stage for subsequent sections on locomotion, movement disorders, and sport science, where nonlinear mechanics provides unique insights into function, variability, and pathology.

#### 2.8.1 Locomotion.

Human locomotion is a complex nonlinear dynamical process governed by the interaction of the central nervous and musculoskeletal systems. The challenge of coordinating multiple joints, muscles, and neural pathways results in movement patterns that are inherently variable, adaptive, and sensitive to perturbations. In fact, the simple act of bipedal walking is an example of a limit cycle. Nonlinear mechanics has been increasingly applied to analyze gait stability, fall prevention, and variability in walking patterns, as it provides a natural basis for describing such high-dimensional interactions.

Nonlinear models of bipedal dynamics have captured critical phenomena such as postural control, joint actuation, and slip events. Muscle–tendon interactions have been represented by lumped parameter systems to study stability and fall prevention [112], while new formulations incorporating foot-slip dynamics improve the realism of gait models [113]. Delay-differential equation models have also been used to examine upright balance, providing insight into feedback and time-delay effects on postural control [114]. These modeling approaches complement empirical studies by enabling controlled perturbations and the prediction of failure modes that are difficult to replicate experimentally.

Nonlinear signal analysis has been used as well. Stride-interval analysis has revealed long-range correlations and fractal dynamics in normal gait, reflecting the intrinsic complexity of locomotor control [115,116]. Multifractality in stride-to-stride fluctuations suggests that healthy locomotion operates across multiple temporal scales, supported by distributed neural and mechanical feedback loops. Importantly, variability is not merely random noise but an organized feature of movement. Reduced variability is associated with rigidity and reduced adaptability, while excessive variability is linked to instability and higher fall risk. Reviews of gait variability emphasize this “optimal state of variability” as a hallmark of healthy movement and a central target for diagnostics and intervention [117,118].

Quantitative system dynamic tools such as detrended fluctuation analysis, multiscale entropy, Lyapunov exponents, and recurrence plots have been employed to characterize this variability and identify subtle impairments. For instance, nonlinear measures can detect early changes in gait associated with aging, neurodegenerative disease such as Parkinson's, or mild injury, *even when conventional spatiotemporal parameters remain within normal limits*. Such methods highlight the potential of nonlinear gait analysis as an accurate biomarker for preclinical diagnostics, long-term monitoring, and evaluation of rehabilitation efficacy.

A deeper mechanistic understanding of gait variability and nonlinear dynamics has broad translational implications. Insights from this work inform rehabilitation after injury, improve mobility in aging populations, support therapy in conditions such as cerebral palsy or Parkinson's disease, and guide the design of efficient bipedal robots or prosthetic devices. By integrating nonlinear science with modern sensing technologies and machine learning, locomotion research is evolving into a powerful framework that bridges biomechanics, clinical diagnostics, and engineering applications, ultimately aiming to reduce fall risk, enhance mobility, and improve quality of life.

#### 2.8.2 Movement Disorders.

Movement disorders such as Parkinson's disease and essential tremor reflect complex interactions between neural and muscular systems. Mechanistic understanding remains incomplete, but nonlinear analyses of motion provide promising avenues for improving diagnosis and treatment.

Quantitative measures of complexity such as multiscale entropy, dominant frequency and amplitude of motion have been applied to differentiate essential tremor from Parkinsonian tremor [119–121]. Such distinctions are clinically important for tailoring therapy. Accelerometry and entropy-based analyses show that tremor signals in Parkinson's disease exhibit reduced complexity and altered scaling dynamics compared to healthy controls [85,122]. Reviews emphasize that structured variability, rather than random noise, characterizes tremor in movement disorders [123]. Wearable sensors and multimodal approaches combining accelerometry and EMG further enhance the ability to monitor motor fluctuations in real-time [124,125].

#### 2.8.3 Sport and Exercise Science.

Research in sport and exercise science often emphasizes descriptive anatomy or empirical training protocols, while mechanistic approaches remain relatively underdeveloped. A deeper understanding of the nonlinear physiological mechanisms underlying adaptation to exercise would provide a stronger scientific basis for training principles, interventions, and injury prevention. Such mechanistic insight would clarify how specific exercise stimuli produce biological changes and help define optimal processes for adaptation [126]. Building on this idea, Levack and Payne advocate for a broader integration of exercise science into interventions, with greater emphasis on physiology-driven modeling rather than trial-and-error methods [127].

A striking example of the knowledge gap is injury susceptibility. Female athletes are known to suffer five to eight times more anterior cruciate ligament injuries than their male counterparts in sports such as soccer, basketball, and volleyball [128,129]. Despite decades of research, the underlying mechanistic causes remain unclear, with hypotheses ranging from hormonal influences on neuromuscular control to biomechanics. The absence of consensus underscores the need for nonlinear, system-level approaches that can integrate multifactorial influences across anatomy, physiology and movement mechanics.

Given these limitations, recent work has increasingly explored complexity science and network physiology as frameworks for exercise research. Balagué et al. [130] propose rethinking exercise physiology through the lens of complex systems, emphasizing the dynamic coupling of cardiovascular, respiratory, neuromuscular and cognitive systems. Nonlinear methods, such as entropy measures, recurrence analysis, and multiscale variability metrics, are beginning to shed light on how performance, fatigue, and recovery emerge from interactions among subsystems rather than isolated responses. This perspective opens new opportunities not only in sports performance but also in sports medicine and rehabilitation, where understanding variability and adaptability is critical for optimizing outcomes while minimizing injuries.

### 2.9 Metabolic System.

Metabolism encompasses all physical and chemical processes that convert or use energy, including breathing, circulation, thermoregulation, muscle contraction, digestion, waste elimination and neural activity [8]. Metabolic dysfunction, most notably diabetes, has reached epidemic proportions, affecting up to 25% of adults globally and representing a major societal challenge.

Mathematical modeling of glucose–insulin dynamics has evolved over several decades, motivated by the need to predict and control highly nonlinear processes. Early compartmental models such as Sorensen's whole-body physiological framework [131] and its refinements [132] provided foundational mechanistic insight. In parallel, classical nonlinear oscillator models, such as the Goodwin model [133] and subsequent work on ultradian (recurrent cycle repeated throughout the day) oscillations of glucose and insulin [134], demonstrated that oscillatory instabilities are intrinsic to metabolic regulation. These insights were synthesized into more comprehensive physiological models, including the multi-organ framework of Guyton and colleagues [135] and later control-oriented models designed for artificial pancreas systems [136]. Nonlinearities such as biphasic insulin release, feedback delays, and parameter sensitivity to patient variability could play decisive roles in diagnosis and therapy.

More recent work has used nonlinear bifurcation analyses to show how small parameter shifts can destabilize metabolic control, such as double Hopf bifurcations in delay-differential glucose models [137]. These methods highlight the diagnostic potential of nonlinear mechanics for predicting transitions from healthy to pathological states. At longer time scales, models capturing beta-cell decline (essential for insulin production) and years-long disease progression [138] and virtual patient populations such as the UVA/Padova simulator [139] from University of Virginia and University of Padova demonstrate how variability and uncertainty can be integrated into translational tools. At the cellular level, nonlinear analyses of mitochondrial dysfunction in aging liver cells [140] and metabolic control analysis based on perturbation theory [141] underscore the diagnostic potential of multiscale nonlinear modeling.

Taken together, these efforts illustrate that nonlinear mechanics and dynamics are not only essential for realistic modeling of metabolism, but also hold promise for early diagnostics, individualized therapy, and the prediction of long-term disease trajectories [142,143].

### 2.10 Respiratory System.

The human lung is a nonlinear, multiscale system whose mechanics determine ventilation and gas exchange. In disease, these nonlinearities are amplified, resulting in severe impairment. Early models focused on lumped mechanical representations of compliance and resistance [144], while more recent approaches integrate multiscale tissue mechanics and systems biology [145]. Nonlinear analyses of lung viscoelasticity demonstrated that stress–strain relations cannot be captured by linear models [146], and inverse modeling frameworks now provide quantitative means to link physiology with mechanics [147,148]. These developments underscore the diagnostic potential of nonlinear approaches for diseases such as asthma, acute respiratory distress syndrome, and chronic obstructive pulmonary disease.

Mechanical ventilation exemplifies how nonlinear interactions between device and physiology shape outcomes. Despite being life-saving, ventilator–lung interactions remain poorly understood, particularly during resuscitation and cardiopulmonary resuscitation (CPR). Recent reviews emphasize that ventilation during CPR is complex, with compressions altering delivered volume and pressure, often compromising efficacy [149,150]. Clinical strategies to optimize for end-tidal CO_2_ [151] and the “six-dial” ventilator method [152] highlight the need for physiologically informed settings, while experimental work shows how chest compressions are nonlinear [153] and can interfere with mechanical ventilation reliability [154]. These studies highlight the importance of nonlinear modeling in optimizing ventilatory support, with direct diagnostic implications for tailoring resuscitation strategies.

Respiratory rhythm generation, reflexes, and interbreath variability also reveal nonlinear signatures. Oscillatory instabilities in rhythm generation [155], reflex interactions [156], and scale-dependent variability in breathing patterns [157,158] have all been documented. Nonlinear metrics such as approximate entropy and correlation dimension have been applied to breathing signals, yielding diagnostic markers for conditions such as sleep apnea [157]. With widespread availability of high-resolution O_2_ and CO_2_ waveforms, there is an excellent opportunity to move these nonlinear analyses modalities from research to clinical diagnostics.

Taken together, these findings show that nonlinear mechanics and dynamics are fundamental to respiratory function. By combining multiscale lung modeling, ventilator–patient interaction analysis, and nonlinear signal processing, diagnostic frameworks could evolve from static pulmonary function measures to dynamic, individualized monitoring tools that would provide critical dynamic diagnostic information to guide both chronic disease management and acute care interventions such as CPR.

### 2.11 Immune Systems.

The immune system is inherently nonlinear, shaped by dynamic interactions between immune cells and target populations such as bacteria, viruses, antigens, and tumor cells [159]. Oscillatory and feedback-driven dynamics are common in both healthy and pathological states [160]. Foundational nonlinear models of viral and immune interactions have been used to demonstrate how feedback loops and parameter sensitivity govern infection control and disease progression [161,162]. Simplified nonlinear ordinary differential equation (ODE) frameworks have captured immune–pathogen interactions [159,163], while delay-differential models highlight how time lags in immune responses shape tumor–immune dynamics [164]. These approaches demonstrate that nonlinear modeling provides valuable diagnostic insights into immune regulation.

Bifurcation analysis has emerged as a central tool for exploring immune dynamics. Early studies showed how cytotoxic T lymphocyte responses to tumor growth undergo local and global bifurcations, predicting disease progression under varying parameters [165,166]. Similar methods have been applied in psychoneuroimmunology, where stress acts as a nonlinear control parameter; models reproduce transitions from stable health to oscillatory or “burn-out” states depending on stress levels [167,168]. Such bifurcation-driven analyses illustrate how nonlinear techniques can reveal diagnostic markers for immune resilience versus breakdown.

Cancer, fundamentally an immune system disease, provides a particularly compelling example of immune system nonlinearity. Tumor–immune interactions involve complex spatiotemporal feedback that cannot be captured by linear analysis [169]. Nonlinear tools such as rising diffusion coefficients from spatial data can anticipate critical transitions in tumor progression, offering potential early warning indicators. Hybrid models, including cellular automata, further capture transient and asymptotic immune–tumor dynamics [170]. Beyond cancer, nonlinear models of antigen discrimination show how the immune system differentiates self from nonself through dynamic thresholds rather than fixed rules [171]. Multiscale approaches integrating T-cell proliferation and turnover dynamics [172,173] demonstrate how cellular feedback scale up to systemic immune responses.

Together, these studies show that nonlinear mechanics and dynamics are fundamental to immune function. By exploiting oscillatory signatures, bifurcations, and spatiotemporal variability, diagnostic frameworks could be developed to identify tipping points in immune responses, stratify disease risk and personalize immunological therapies.

### 2.12 Neural Systems.

**Fig. 5:**
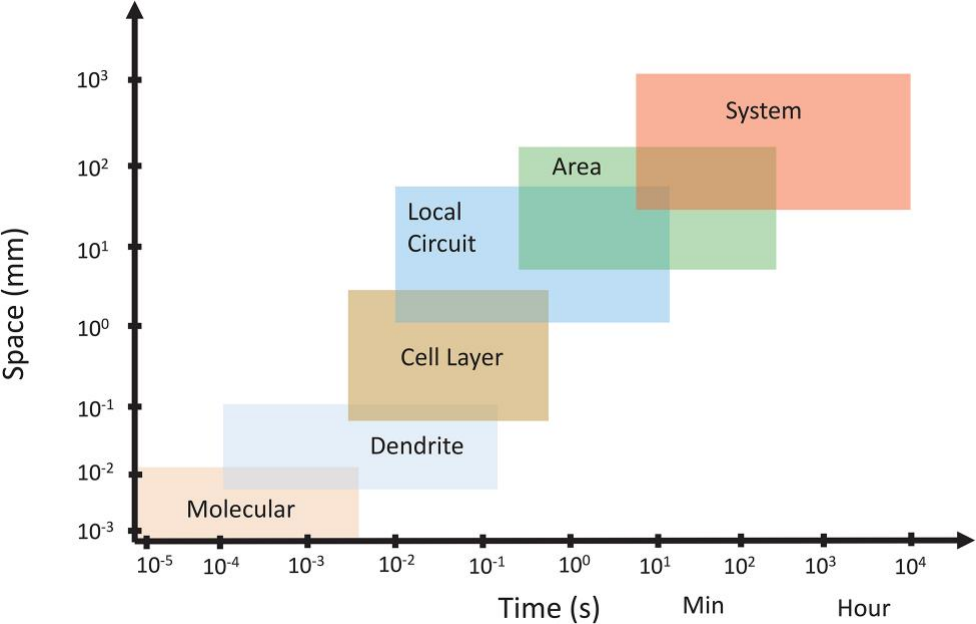
Temporal and spatial scales of organization in the nervous system. We can observe that at the smaller spatial scales, the time scales are also smaller (there is a correlation between spatial and temporal scales of the system). Arguably, the brain operates on multiple spatial and temporal scales more than any other organ. Adapted from Ref. [178]. Used with permission.

Neural systems, from single neurons to large-scale brain networks, are inherently nonlinear and stretch across multiple scales (Fig. 5). The mechanistic analysis of neural activity started with a historic Nobel prize-winning study of the squid giant axon for which the revolutionary Hodgkin–Huxley model was developed and validated [174]. This model is a set of nonlinear differential equations that describes how action potentials in the neurons are initiated and propagated. This model approximates the electrical characteristics of excitable cells such as neurons and cardiac cells and was first used to describe the ionic mechanisms underlying the initiation and propagation of action potentials in the single neuron. Later, population-level models such as Wilson–Cowan [175] captured emergent oscillations from excitatory–inhibitory feedback. Building on these foundations, nonlinear neurodynamics has been developed as a unifying framework for understanding brain function [176,177]. These models highlight how rich collective phenomena, such as synchronization, oscillatory bursts, and critical transitions, emerge from relatively simple nonlinear interactions.

Electroencephalography (EEG) and magneto-encephalography have been central in applying nonlinear signal analysis to clinical diagnostics. Methods such as correlation dimension, Lyapunov exponents, entropy measures, detrended fluctuation analysis, wavelet decomposition, and multifractal formalisms have revealed that neural activity often resides on low-dimensional attractors and exhibits scale-dependent variability [179]. Nonlinear signal analysis has uncovered dynamic regimes that are invisible to conventional spectral techniques, making it particularly useful for differentiating healthy and pathological brain states. For example, nonlinear coupling metrics have been shown to reflect long-range synchronization across brain regions, which is often impaired in disease.

These methods have shown diagnostic promise across a wide spectrum of disorders. Epilepsy has three phases, in the form of beginning (pre-ictal), middle (ictal), and end (postictal) stages, with intense electrical activity occurring in the brain in the ictal stage [180]. As of today, the mechanistic cause of epilepsy is not completely understood. The seizures are frequently modeled as low-dimensional oscillatory instabilities, with nonlinear metrics aiding in pre-ictal detection and forecasting [181,182]. Phase synchronization and nonlinear Granger causality [183,184] have been used to map seizure propagation, providing potential tools for surgical planning.

In sleep research, entropy-based analyses and complexity measures distinguish between stages of sleep and detect abnormalities in conditions such as sleep apnea, narcolepsy, and rapid eye movement behavior disorder [185]. Nonlinear approaches have also been applied to circadian rhythms, where fractal scaling of EEG and respiratory signals reveals altered dynamics in patients with sleep fragmentation.

In dementia and related cognitive disorders, reductions in EEG complexity and changes in oscillatory organization have been consistently reported. Decreases in multiscale entropy and alterations in nonlinear synchronization patterns have been linked to Alzheimer's disease and mild cognitive impairment, serving as candidate biomarkers for disease progression [186–188].

In Parkinson's disease, nonlinear EEG and magnetoencephalography analyses have revealed reduced signal complexity, impaired synchronization, and abnormal transitions between oscillatory states [189,190]. Such nonlinear markers are being investigated as potential biomarkers to distinguish disease stages and monitor therapeutic response.

Taken together, these findings demonstrate that nonlinear mechanics and dynamics provide a powerful diagnostic lens for neural systems. By characterizing brain activity in terms of attractors, bifurcations, synchronization, and critical transitions, nonlinear methods move beyond linear correlations and static spectral features. This enables the development of sensitive and dynamic biomarkers capable of tracking disease onset, progression, and therapeutic response across a wide range of brain disorders.

### 2.13 Summary.

**Fig. 6:**
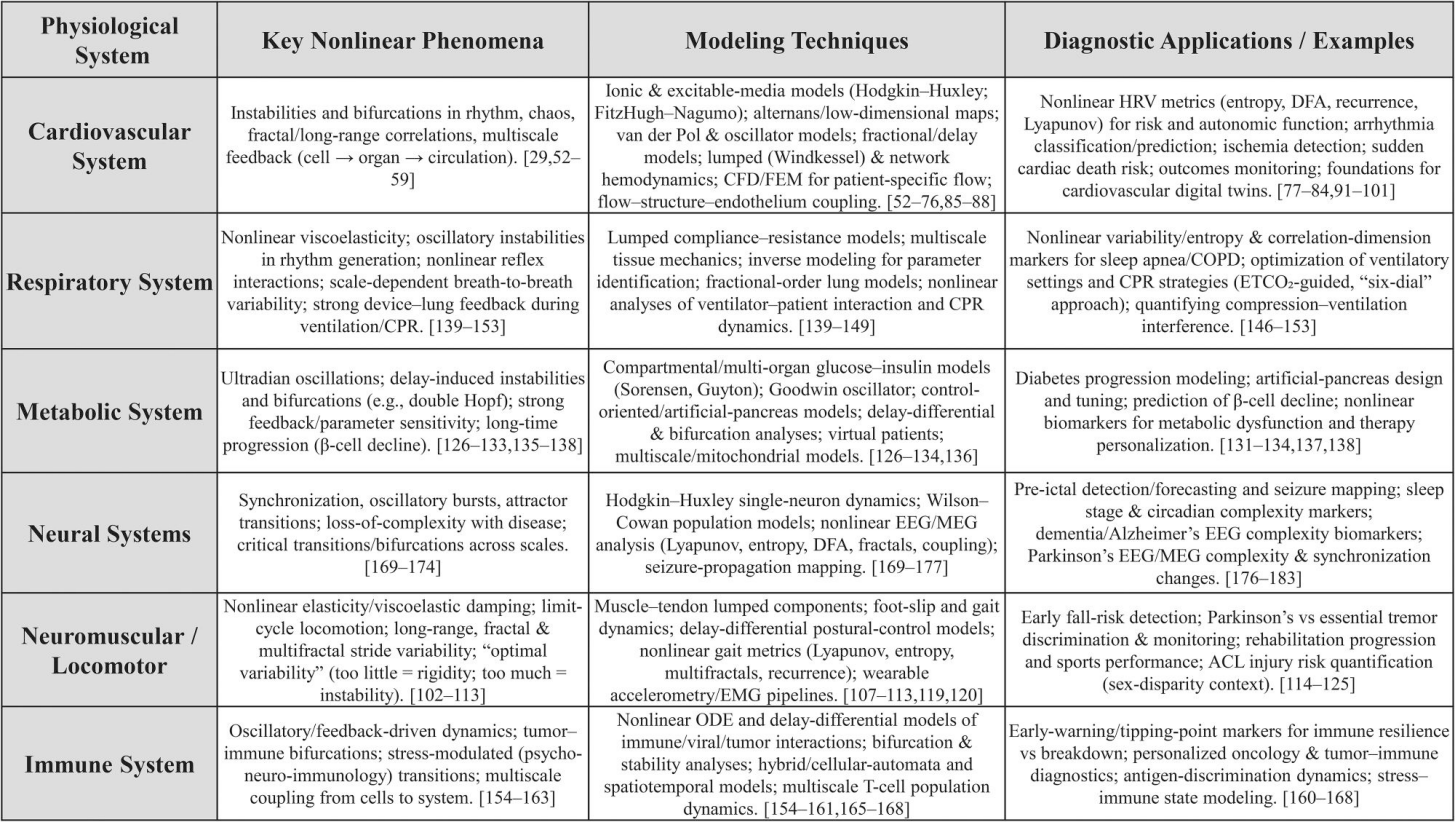
Summary of nonlinear mechanics in the human body: systems, phenomena, techniques and applications

In summary, there has been a spectrum of studies on nonlinear phenomena in cardiovascular, respiratory, metabolic, neural, and immune systems. This is compactly summarized in Fig. 6. This body of work is impressive and has significant potential to be applied for medical diagnostics when used in conjunction with data-based techniques as outlined in the following sections. However, we would like to emphasize that there persists a continuing need for further investigations to obtain mechanistic insights into the various systems in the body, in particular, to uncover physiologically important nonlinear phenomena.

## 3 The Diagnostics Problem

### 3.1 Artificial Intelligence and Machine Learning Approaches.

Currently, medical diagnostics is overwhelmingly in the hands of the medical practitioner (the “expert”) who collects data using instruments, etc., as we discussed in the Introduction. Increasingly, the advent of artificial intelligence (AI) has brought in a lot of promises and, in fact, “an irrational exuberance”. In both cases, the principal task is the conversion of *information* to generalizable *knowledge* with the least error in the presence of uncertainty and variability. In the following, we discuss the current state of AI tools to accomplish this task.

Artificial Intelligence is a catch-all term for automating mental tasks so that we can replace humans with computers to perform those tasks. AI's recent rise was fueled by exponential improvements in processing speed and by falling costs in sensing leading to an explosion of information, the so-called big data phenomenon. Machine learning (ML), in particular, is a popular approach to AI with a growing body of algorithms that attempt to “learn” the relationship of measured inputs to outputs by minimizing an error metric while recognizing the statistical spread in real data.

Over the past decade there has been a high level of enthusiasm for machine learning fed by the happy confluence of new research into algorithms, dramatically increased processing power (with quantum computing looming on the horizon) and a large-scale collection of rich and diverse data. Although it is too early to foretell the future evolution of this technology, it is almost certain that we will see many success stories from AI in medical diagnostics although the pure data-based algorithms are bound by some hard-to-resolve limitations as explained below.

**Fig. 7:**
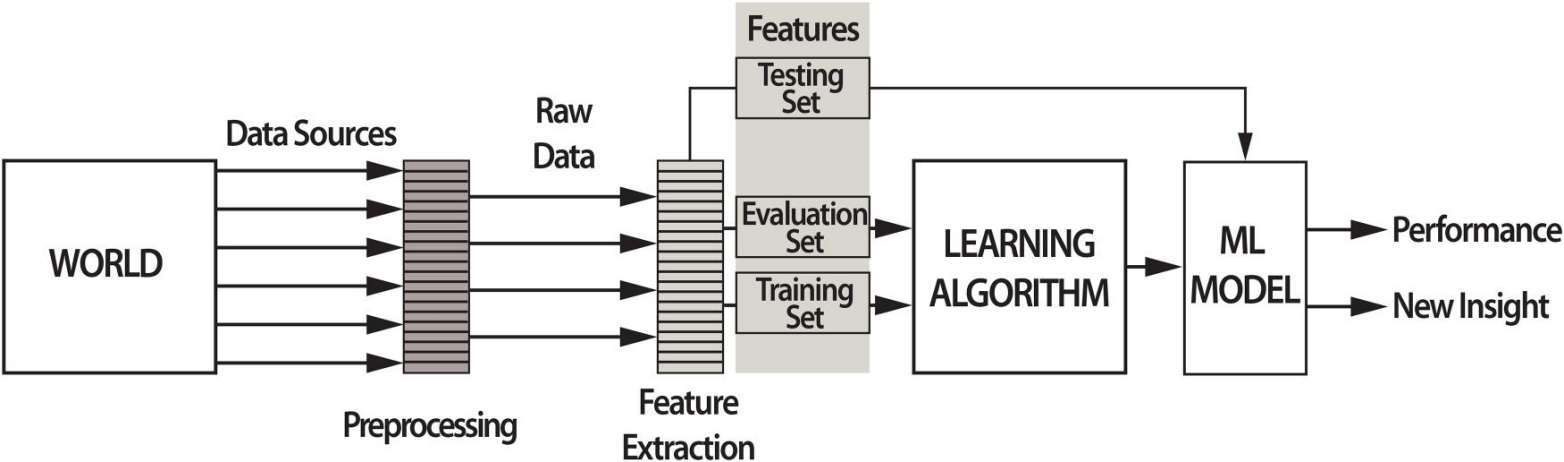
A high-level overview of the standard ML process

In a typical ML structure, an optimizing algorithm aims to establish a meaningful mathematical function that maps different observations of an instance or a sample to its outcome or meaning. The functions are referred to as models and the descriptions of the observations or the instances as data. Figure 7 illustrates the traditional ML process.

Recorded observations often require some preprocessing such as data cleansing, data merging, imputation, synchronizing, and filtering before it can be used in an instance domain. In the feature extraction process, the raw data is mathematically mapped from the instance domain to the feature domain, translating the raw data into numeric quantities and making it suitable for the learning algorithm. Given the huge volume of the medical data, which can potentially be used for diagnostics, it is important to reduce the size of the raw data, and capture the essence of it (i.e., the information). The feature extraction process facilitates this matter. A *feature* is a relevant and informative aspect of the dataset (e.g., the peak value of the blood pressure signal). We need features, as compact abstractions of the raw data, in order to be able to create a model, capable of generalizing the learned patterns. In addition, the fundamental principle of the “curse of dimensionality” [191,192] dictates that it is always better to have fewer *good* features than a lot of *bad* ones. This concept has led to the development of feature set optimization, in which one starts with a pool of features and then tries to eliminate the features that are not very informative.

The term *model* here refers to a mathematical relationship obtained from data using a learning algorithm. The ML model type is determined by both the nature of this relationship and the specific learning algorithm employed. Additionally, the task at hand and the characteristics of the available data are often the determining factors for the ML model type required to accomplish a given objective. As a result, there exists a wide variety of ML models suited to different tasks and learning paradigms; yet, the fundamental steps of the ML process remain broadly comparable.

*Learning* or *training* refers to the process of utilizing algorithms to generate models from data. The data utilized in the training phase are referred to as the *training set*, which is often divided into training and *validation* subsets throughout the learning process (Fig. 7). Because the learning agent attempts to construct a function based on underlying rules within the data, this function is generally referred to as a *hypothesis*, while the true underlying rules are referred to as *facts* or *ground truth*. The agent's goal is therefore to discover or approximate these ground truths by exploring its environment specified by the training set. The learning step consists of numerous iterative approximations until the final learned model satisfies predefined performance criteria.

In the context of nonlinear and physics-informed modeling, these data subsets play complementary but distinct roles. The *validation set* is used during model development for hyperparameter tuning, model architecture selection, and early stopping procedures that guide optimization while minimizing overfitting to the training data. Validation results inform refinement of the model and regularization parameters, but do not contribute directly to weight updates. By contrast, the *test set* is completely held out from both training and validation stages and is evaluated only once after model development to assess generalization performance on unseen physiological data. This separation ensures that the reported diagnostic accuracy reflects the model's ability to capture underlying dynamical relationships rather than to reproduce specific trajectories from the training data.

The process of assessing the learned model is referred to as *testing*, and the samples to be evaluated by the model are new instances from the test set that the learned model has never encountered before. In this way, the test phase provides an unbiased measure of predictive generalization, an essential criterion for evaluating nonlinear or physics-informed learning systems intended for medical diagnostics.

**Fig. 8:**
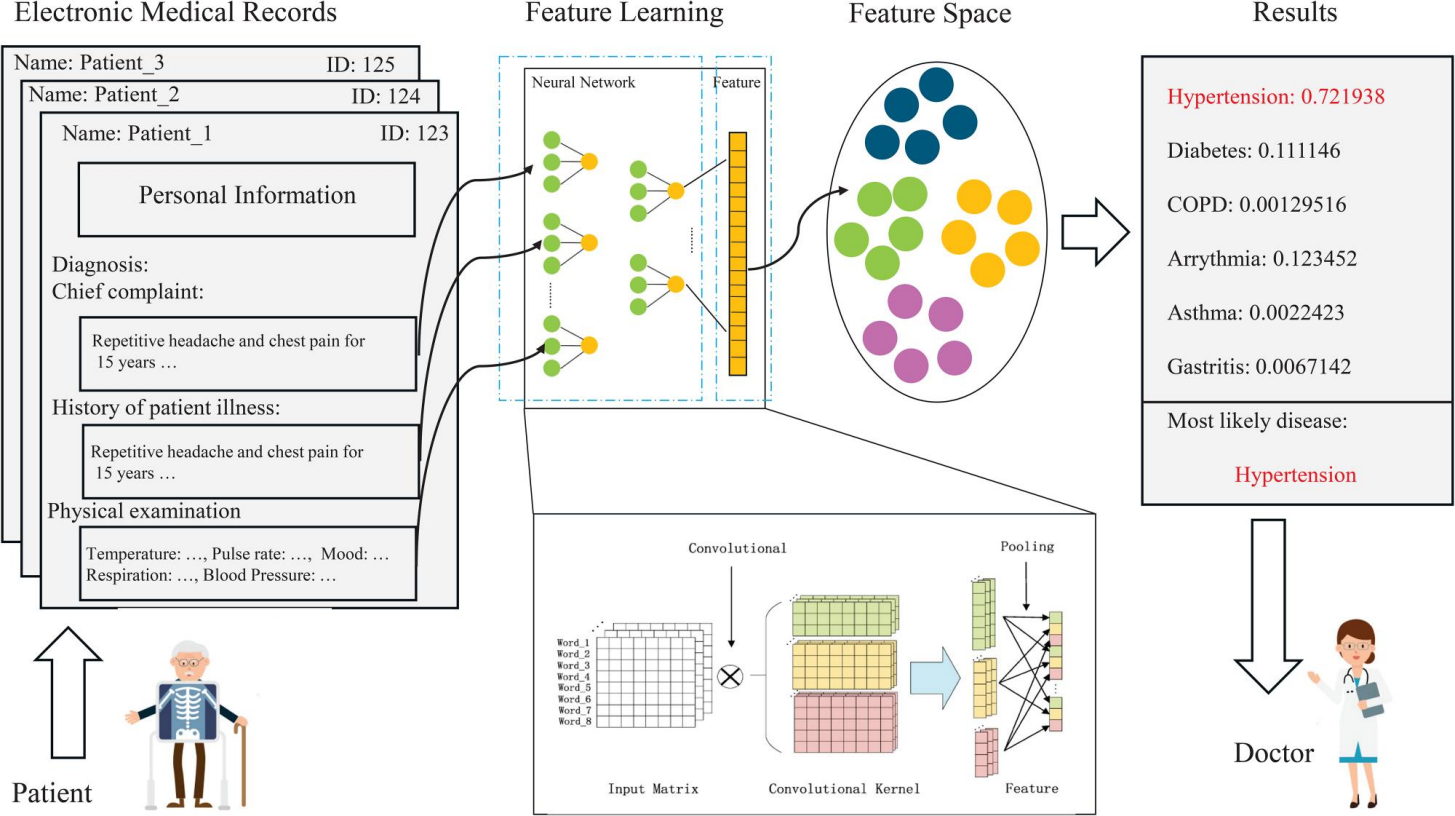
A hypothetical CNN architecture for clinical application. Adapted from Ref. [195]. Used with permission.

In contrast to the traditional machine learning workflow shown in Fig. 7, where feature extraction constitutes a separate preprocessing stage, deep learning frameworks integrate feature learning and classification within a unified architecture. Modern approaches, such as convolutional (CNN), recurrent (RNN), and transformer-based neural networks, enable hierarchical representation learning directly from raw or minimally processed data. This end-to-end optimization has been applied to biomedical signal and image analysis, allowing models to discover complex, multiscale features that are difficult to engineer manually [193,194]. A typical deep learning architecture is shown in Fig. 8.

Artificial intelligence (deep learning, in particular) has already had major impact in radiology, pathology, and dermatology, largely because those domains involve high-quality static images with relatively clear diagnostic endpoints. By contrast, progress in dynamic physiological signals has been slower. Most AI applications to time-series data still rely on pattern recognition in EKG or EEG waveforms without accounting for the nonlinear, feedback-driven nature of the underlying physiology. This gap has left many models brittle, poorly generalizable, and clinically unconvincing. Current systems often excel on benchmark datasets yet fail at the bedside, where signals are noisy, nonstationary, multimodal, and profoundly patient-specific [196,197].

In biomedical imaging, nonlinear dynamics provide a useful lens for interpreting structural complexity and physiological meaning. Image features in modalities such as radiology, histopathology, and biomedical microscopy often arise from underlying dynamical processes including tissue deformation, perfusion, diffusion, and growth. Accordingly, nonlinear descriptors such as fractal dimension, entropy, and texture complexity capture diagnostically relevant information that reflects the system's spatiotemporal organization [198,199]. Recent advances in physics-informed and dynamics-augmented vision models extend this principle by embedding biomechanical or diffusion-based priors within convolutional and transformer architectures, thereby linking spatial image complexity with the temporal evolution of physiological processes [200,201].

A major shortcoming of the AI/ML approach in general is that many models discard the temporal richness of physiologic signals, reducing them to static features or summary statistics. This ignores the fact that diagnostic information is often encoded in the *dynamics*: variability, coupling, synchronization, and regime changes across multiple time scales. Attempts to apply black-box models without mechanistic grounding risk producing uninterpretable outputs, eroding clinician trust, and amplifying biases. Moreover, label scarcity is a persistent challenge: high-quality annotations for long waveforms are costly, inconsistent, and often infeasible to obtain at scale [202,203].

To overcome these barriers, two complementary strategies have emerged. (i) *Mechanistic–feature pipelines*: nonlinear features such as multiscale entropy, DFA scaling exponents, recurrence metrics, Lyapunov exponents, and phase–amplitude coupling provide interpretable summaries of stability and complexity [83,85,204]. These approaches are data-efficient and align with known physiology, but may miss richer structure. (ii) *Sequence models*: CNNs, TCNs (temporal convolutional networks), RNNs, Transformers, and latent state–space models can learn directly from raw waveforms, capturing long-range dependencies and nonlinear interactions [205,206]. Hybrid approaches that embed mechanistic priors or physics-informed loss terms inside deep sequence models are especially promising, as they combine interpretability with representation power. However, they increase the black box nature of the ML algorithms further reducing trustability.

More advanced frameworks include neural ordinary differential equations/stochastic differential equations (ODE/SDE) and physics-informed neural networks (PINNs), which integrate differential-equation structure into learning. These approaches allow models to respect conservation laws, delays, and constitutive relationships, yielding more robust generalization and physiologically meaningful latent states [201,207]. These methods are further discussed in the next section. Fractional-order models extend this further by embedding memory and viscoelastic effects that are prominent in cardio–respiratory systems. However, these methods are computationally demanding and not yet validated in large-scale clinical workflows.

Another persistent issue is the gap between research benchmarks and bedside deployment. Many published models are trained and tested on carefully curated datasets with minimal noise, limited demographics, and artificially clean signals. In real clinical environments, artifacts, missing data, and population heterogeneity dominate. Few models incorporate uncertainty quantification, leading to overconfident predictions that are unsafe for decision support. Bayesian deep learning [208] and conformal prediction [209] provide possible solutions, but remain rarely applied in prospective clinical studies.

Despite these shortcomings, AI has shown that dynamic modeling can add real value in several domains. Arrhythmia detection from EKG [210], modeling cardiac regulation mechanisms [211], seizure forecasting from EEG, prediction of neurological damage in preterm infants [212], and risk prediction in intensive care unit (ICU) telemetry [213] are areas where temporal dynamics outperform static snapshots [214,215]. Respiratory failure alerts, chronic obstructive pulmonary disease exacerbation monitoring, and multimodal sepsis predictors provide further examples. In each case, the diagnostic signal lies not in a single value but in the evolving waveform: its variability, coupling, and transitions between regimes.

As discussed in the motivating example in Introduction, CPR continues to be a challenge given its dismal effectiveness (2–15% survival rates and poor neurological outcomes in those who do survive cardiac arrest). ML techniques have proved to be somewhat useful in nudging our understanding of the CPR process. In this problem, researchers have used dynamic modeling and developed algorithms that were guided by physician insight. Successes include automatic and real-time classification of etiology of the cardiac arrest (asphyxia versus ventricular fibrillation), a key determinant of the CPR delivery protocol that determines survival [216]. The critically important task is to convert CPR from a guideline-centric procedure to a patient-centric one, in the sense that the treatment will follow a diagnostic algorithm that relies on the *dynamic condition* of the patient [217].

While AI has demonstrated proof-of-concept in analyzing dynamic physiologic signals, its clinical impact has been limited by noise, label scarcity, lack of mechanistic integration, limited generalizability, and insufficient attention to nonlinearity. Addressing these challenges requires models that respect physiological structure, quantify uncertainty, and prove their robustness in prospective, real-world settings. Only then can AI move from narrow benchmarks to reliable, nonlinear, dynamic diagnostics at the bedside.

Finally, the issue of the trustworthiness of ML is becoming increasingly of concern because of the black box nature of the system. In addition to privacy and security concerns, the lack of transparency is likely to limit adoption by the medical community. Relegating life or death decisions to machines, except for routine matters, seems especially risky to many clinicians and researchers alike [15]. In summary, notwithstanding the accomplishments and potential future achievements, it is our opinion that pure data-based AI methodologies will plateau simply because of some of these issues.

## 4 Discussion

Medical diagnostics represents a fundamentally epistemological challenge: an effort to infer the underlying state of a complex, nonlinear system from limited, noisy observations. Historically, this process has relied primarily on clinical expertise, shaped by accumulated medical knowledge and intuition. Yet, as physiological data have become increasingly high-dimensional and dynamic, the limits of human inference have become apparent. In parallel, data-driven approaches such as Machine Learning and Artificial Intelligence have demonstrated remarkable pattern-recognition capabilities, while nonlinear mechanics offers the governing physical principles that explain how biological systems behave and evolve. A comprehensive diagnostic framework must therefore integrate these three perspectives.

Each paradigm contributes distinct advantages and inherent limitations. Clinical reasoning embodies physiological understanding and experiential judgment but is constrained by cognitive and perceptual limits when interpreting complex temporal patterns. Purely data-driven algorithms excel at extracting structure from massive datasets, but they often lack interpretability, depend heavily on large labeled samples, and fail to encode underlying causal or nonlinear mechanisms [218,219]. Physics-based models, conversely, capture mechanistic interactions and feedback structure but are necessarily simplified and computationally demanding, limiting their predictive ability to generic phenomena. The convergence of these methodologies – human expertise, data-driven inference, and nonlinear mechanics – defines the emerging frontier of diagnostic science.

Physics- and physiology-informed machine learning provides a natural vehicle for such synthesis [200]. By embedding physiological constraints and nonlinear dynamics directly into learning architectures, these methods improve generalization, interpretability, and trustworthiness. Moreover, clinical expertise can be used to define priors, select meaningful features, or constrain solution space and inference, thereby aligning algorithmic outputs with physiological plausibility. In this way, a hybrid framework can exploit the interpretive power of physics, the scalability of data-driven learning, and the contextual insight of clinical reasoning.

One of the most potentially productive pathways for this integration lies in the development of *digital twins*. By embedding nonlinear physiologic mechanisms, e.g., nonlinear resonances [220], hysteresis, bifurcations, and multiscale coupling, into learning algorithms, digital twins provide a structural backbone that ensures coherence between mechanistic understanding and data assimilation. The following subsections summarize the analytical, computational, and multiscale foundations that underpin this paradigm.

### 4.1 Digital Twins.

The concept of the digital twin, originally developed in aerospace [221] and manufacturing [222], has the rich potential to be applied in medicine. A digital twin is a dynamically updated computational replica of a physical system that continuously assimilates empirical data [223]. In healthcare, it represents a patient-specific model that integrates physiologic signals, mechanistic equations, and adaptive inference in real-time [224–226].

Conventional diagnostics rely on consensus-based, population-level guidelines, which, while invaluable, cannot accommodate the vast interindividual variability in physiology and regulation. Digital twins can overcome this limitation by embedding nonlinear mechanics within adaptive computational frameworks capable of representing both universal principles and individual differences. Physiological processes such as cardiac conduction, neural oscillation and hemodynamic regulation exhibit nonlinearities including hysteresis, resonance, intermittency and bifurcation; such behaviors cannot be captured by linear or purely statistical models. Incorporating these mechanisms enables realistic simulation and prediction of patient-specific responses [227–229]. For example, nonlinear arterial elasticity, viscoelastic damping and feedback control loops are essential for predictive cardiovascular modeling.

**Fig. 9:**
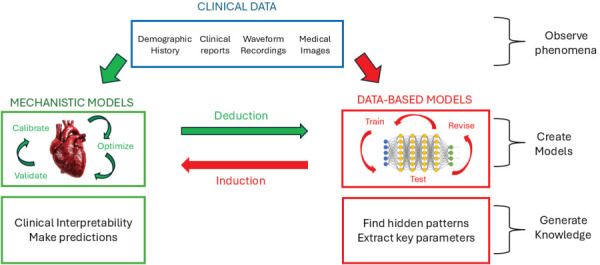
Conceptual illustration of a cardiovascular digital twin, integrating nonlinear mechanistic models with data-driven inference for real-time physiological prediction. Adapted from Ref. [227]. Used with permission.

The power of digital twins lies in their fusion of mechanistic and data-driven paradigms. Mechanistic models provide interpretability and generalizability, while ML models personalize inference through continuous data assimilation [230,231]. Figure 9 illustrates this integration.

Despite its promise, significant challenges remain before digital twins can be routinely implemented in clinical workflows [232]. These include the need for high-fidelity simulations, translational validation in animal and human studies, and rigorous uncertainty quantification (UQ) for regulatory acceptance [81,233,234]. Because nonlinear systems exhibit sensitivity to small perturbations, UQ is critical to ensure robustness and interpretability. Modular architectures such as hub-and-spoke configurations and multifidelity coupling have been proposed to integrate diverse component models while maintaining coherence [235]. Without such safeguards, digital twins risk producing artifacts rather than actionable insights.

### 4.2 Data Abundance, Scarcity, and Label Limitations. 

Modern medicine is characterized by a paradoxical duality of data availability. Certain domains, most notably genomics, medical imaging, and wearable sensing, generate massive and rapidly expanding datasets [236–238]. Yet many clinical contexts remain fundamentally *data-scarce*, constrained by acquisition cost, privacy regulations, ethical limitations, and the rarity of specific disease conditions [239,240]. This imbalance highlights the need for modeling approaches that can extract maximal insight from minimal data. Physics-informed and nonlinear frameworks are particularly suited to this challenge, as they embed mechanistic structure such as conservation laws, stability constraints, and dynamical priors, directly into inference processes, thereby reducing dependence on large, exhaustively labeled datasets [200,241]. By exploiting these embedded physical constraints, such methods can achieve robust generalization and interpretability even in small-sample or heterogeneous clinical regimes.

Recent advances in generative artificial intelligence provide powerful means for addressing data scarcity in biomedical applications. Variational auto-encoders (VAEs), generative adversarial networks (GANs), and diffusion models enable the synthesis and augmentation of realistic physiological and imaging data, enhancing model robustness under limited sample conditions [242,243]. In addition to data generation, emerging fusion architectures integrate physics-based and data-driven approaches at the output level through parallel, sequential, or hybrid configurations. These frameworks combine mechanistic models such as physics-informed neural networks (PINNs) or digital twins with deep learning networks, thereby unifying predictive generalization with physical and physiological interpretability.

A closely related issue is *label scarcity*, which arises because expert annotation in medicine is costly, subjective, and inconsistent across institutions. Nonlinear modeling alleviates this limitation by encoding physical laws and dynamical invariants as implicit supervisory signals. Physics-informed neural networks (PINNs) and related architectures integrate conservation and stability constraints directly into loss functions, enabling meaningful learning from unlabeled or sparsely annotated data [244–246]. In parallel, advances in self-supervised and contrastive learning exploit the temporal and structural richness of physiological data through *dynamical augmentations* predicting future states, reconstructing attractor geometry, or contrasting perturbed trajectories, to derive pseudo-labels from intrinsic system behavior [247–249]. Together, these developments demonstrate that combining mechanistic priors with self-supervised objectives provides a principled and scalable pathway for interpretable learning when labeled data is limited.

### 4.3 Neural Ordinary Differential Equations, Neural SDEs, and Physics-Informed Networks.

Neural ordinary differential equations (neural ODEs) and their stochastic counterparts (neural SDEs) extend deep learning into continuous-time dynamics, offering a natural bridge between mechanistic and data-driven inference. In these models, hidden-state evolution is governed by a learnable differential operator rather than discrete layers, enabling direct approximation of underlying dynamical laws. Neural ODEs excel in smooth, low-dimensional systems, while Neural SDEs capture diffusion-dominated or noise-driven phenomena by parameterizing both drift and stochastic terms [250,251]. This yields interpretability, temporal coherence and parameter efficiency in modeling physiological time series.

Physics-informed neural networks (PINNs) generalize these approaches by embedding known governing equations into the learning process, minimizing residuals of differential operators and measurement constraints simultaneously [200,241]. Applications span cardiovascular flow modeling, perfusion imaging, and neuroelectric field estimation [244–246]. The principal advantages of these models are reduced data requirements and improved generalization through mechanistic regularization. However, they face challenges related to computational cost, stiffness in learned dynamics and sensitivity to incomplete or uncertain physical formulations. Current research in adjoint-based solvers, adaptive sampling and multifidelity training could expand their clinical utility.

### 4.4 Systems Biology and Multiscale Dynamics.

The same nonlinear principles that govern organ-level regulation also apply at the molecular and cellular scales. Systems biology increasingly employs physics-informed frameworks to model biochemical feedback, oscillation and stability. Recent promising developments include *AI-Aristotle*, a gray-box identification framework combining mechanistic reaction models with ML to infer hidden regulatory interactions from sparse data [252]; *Systems-informed neural networks*, that embed conservation laws and reaction kinetics within PINNs [253]; and, sparse-regression techniques can recover coupling structure even under noise [254,255]. These frameworks establish a continuum between molecular networks and macroscopic physiology.

Physiological regulation emerges through hierarchical coupling across scales from molecular signaling to organ-level behavior. Nonlinear mechanics provides a natural formalism for this integration using coupled differential systems, multiscale networks and homogenization techniques. Cardiac, neural and motor systems display cross-scale feedback that produces the fractal variability characteristic of healthy dynamics [44,48,54]. Extending digital twins across scales would allow molecular kinetics, organ mechanics and behavioral feedback to coexist within unified predictive models.

### 4.5 Molecular Dynamics as a Frontier of Diagnostic Modeling.

At the molecular scale, molecular dynamics (MD) simulations reveal atomistic fluctuations and energy-landscape transitions that embody hallmark nonlinear phenomena such as sensitivity to initial conditions and metastable switching. Quantifying these trajectories using nonlinear measures such as conformational entropy, energy topology, and collective-mode dynamics, which link molecular processes to systemic stability. Recent work introduced *Dynamicasome*, an MD-guided and AI-driven pathogenicity prediction framework mapping the dynamical signatures of genetic mutations [256]. Such approaches bridge molecular and clinical domains, demonstrating that the same nonlinear principles unify structure, function, and pathology across biological hierarchies.

### 4.6 Synthesis: Toward a Unified Dynamic Diagnostics.

The preceding sections collectively support a unified view of diagnostics as dynamical inference. Health corresponds to organized complexity — adaptive, fractal, and resilient — whereas disease represents a transition in system stability and coupling. In this paradigm, the primary object of diagnosis is the trajectory of a living system within its high-dimensional state space, not static measurements or thresholds. Physiologically observable signals such as heart rate, arterial blood pressure, neural oscillations and metabolic flux trace projections of this trajectory. Nonlinear analysis reconstructs these dynamics, revealing early signatures of instability and loss of adaptability.

Integrating physics-informed learning and digital twins operationalizes this view: diagnosis becomes the estimation of evolving dynamical equations rather than classification of snapshots. Multiscale coupling ensures coherence from moleorgans to organ, enabling early detection of transitions that preceded clinical manifestation. Variability, in this context, is reinterpreted not as noise but as a marker of vitality, complexity signals adaptability. Nonlinear diagnostics would thus transform the clinician's task from labeling disease to interpreting dynamic organization.

### 4.7 Outlook and Future Directions.

The proposed framework of nonlinear diagnostics invites a transformation in how medicine measures, predicts, and manages disease. Several directions define the path forward.

First, developing integrated computational infrastructures that link high-fidelity sensing, analytics (likely on the cloud), and physics-informed digital twins would enable continuous monitoring of physiological dynamics. Such systems would provide early detection of instability, personalized treatment optimization, and adaptive therapeutic control.

Second, robust uncertainty quantification and validation are essential for clinical translation. Because nonlinear models are sensitive to parameter variation, explicit representation of epistemic and aleatoric uncertainty is critical for reliability and regulatory confidence [49]. Ethical governance must evolve concurrently to address data ownership, transparency, and accountability.

Third, progress depends on interdisciplinary ecosystems that bring together clinicians, engineers, physicists and computer scientists. Training programs that unite physiology, mechanics and AI will be vital for cultivating expertise in dynamic diagnostics. Translational centers combining modeling, ML and data pipelines can accelerate feedback between research and clinical practice.

Finally, this paradigm implies a profound philosophical shift—from a static taxonomy of disease to a dynamic science of health. Diagnostics grounded in nonlinear mechanics view variability as evidence of resilience rather than disorder. As computation, sensing, and modeling converge, the synthesis of nonlinear dynamics, artificial intelligence, and physiology points to a path to redefine diagnostics as the study of evolving systems, one where prediction, personalization and prevention would be unified.

## 5 Conclusion

This paper has argued that nonlinear mechanics and dynamics are not mere curious aspects of physiology, but rather fundamental principles that govern every level of human function. From cellular excitability and electrophysiology to hemodynamics, respiration, metabolism, locomotion, and neural control, nonlinearities such as bifurcations, chaos, fractal scaling and multiscale couplings are central to both health and disease. By ignoring these dynamics, current diagnostic practice remains fundamentally limited, being still largely reliant on guideline-based heuristics and linear statistical models. As it has been noted, Life itself expresses fractal dynamics that degrade with disease and aging, underscoring the essential diagnostic value of nonlinear analysis [30].

We have reviewed evidence across major physiologic subsystems showing how nonlinear dynamics can enrich mechanistic understanding and yield diagnostic markers inaccessible to linear approaches. At the same time, we have highlighted the shortcomings of relying exclusively on physician expertise or purely data-driven AI models. Human processing power, though adaptive and wise, is not equipped to parse and analyze high-dimensional nonlinear signals without assistance from computing tools. Machine learning, while powerful at pattern recognition, often lacks interpretability, generalizability, and grounding in physiology. Physics-based modeling provides mechanistic clarity but struggles with patient-specific variability and the full complexity of human physiology. Each perspective, in isolation, is insufficient.

The synthesis of these approaches offers the greatest promise. Physics-informed machine learning, digital twin frameworks and hybrid models can integrate nonlinear mechanistic structure with patient-specific data and clinical intuition. Such systems have the potential to deliver adaptive, personalized and predictive diagnostics that transcend the current limitations of guideline-driven medicine. The vision is a transformation from reactive treatment after disease has progressed, to pro-active management guided by dynamic, mechanistic insight. This convergence of human and artificial intelligence would represent a new era of medicine.

Realizing this vision will require translational studies, clinical trials, and rigorous validation. Challenges include data heterogeneity, computational latency, regulatory acceptance, and the need for modular, scalable architectures with robust uncertainty quantification. Yet, these are surmountable obstacles, given the rapid advances in sensing, high-performance computing, and AI. Just as engineering harnessed nonlinear mechanics to design aircraft, spacecraft and nanodevices, medicine can now harness these tools to redesign diagnostics and treatment for the most complex system of all: the human body. The digital twin, already being explored in cardiovascular medicine, exemplifies this transformation by fusing nonlinear mechanics with real-time patient data to enable precision diagnostics and therapy.

In conclusion, nonlinear mechanics provides the scientific backbone, AI and machine learning provide adaptive data-driven power, and physicians provide wisdom and context. Together, they point toward a future where diagnostic errors are reduced, interventions are personalized, and medicine evolves into a truly predictive science. The integration of nonlinear dynamics into diagnostics is not simply a technical refinement; rather, it represents a paradigm shift in how we understand, monitor and care for human life.

## References

[1] Erdi, P., 2008, Complexity Explained, 1st ed., Springer-Verlag, Berlin Heidelberg.

[2] West, B. J., 2006, Where Medicine Went Wrong: Rediscovering the Path to Complexity, Vol. 11, World Scientific, Singapore. 10.1142/6175

[3] Buckley, U., and Shivkumar, K., 2016, “Stress-Induced Cardiac Arrhythmias: The Heart-Brain Interaction,” Trends Cardiovascular Medicine, 26(1), pp. 78–80. 10.1016/j.tcm.2015.05.001 PMC4662914 26051207

[4] Arbib, M., Erdi, P., and Szentagothai, J., 1997, Neural Organization: Structure, Function and Dynamics, MIT Press, Cambridge, MA.https://mitpress.mit.edu/9780262526418/neural-organization/

[5] Chalmers, D. J., 1996, The Conscious Mind: In Search of a Fundamental Theory, Oxford University Press, New York.https://global.oup.com/academic/product/theconscious-mind-9780195117899?cc=in&lang=en&

[6] Montgomery, K., 2012, How Doctors Think: Clinical Judgment and the Practice of Medicine, Oxford University Press, New York.https://www.researchgate.net/profile/Kevin-Barraclough/publication/25237818_How_Doctors_Think_Clinical_Judgment_and_the_Practice_of_Medicine/links/54f976c30cf210398e989dff/How-Doctors-Think-Clinical-Judgment-and-the-Practice-of-Medicine.pdf

[7] Eikeland, O., 2008, The Ways of Aristotle: Aristotelian Phrónêsis, Aristotelian Philosophy of Dialogue, and Action Research (Studies in Vocational and Continuing Education), Vol. 5, Peter Lang, Bern, Switzerland.

[8] U.S National Library of Medicine, 2022, “Metabolism: Trusted Health Information for You,” U.S National Library of Medicine, Bethesda, MD, accessed Nov. 1, 2025, https://medlineplus.gov/ency/article/002257.htm

[9] Saber Tehrani, A. S., Lee, H., Mathews, S. C., Shore, A., Makary, M. A., Pronovost, P. J., and Newman-Toker, D. E., 2013, “25-Year Summary of US Malpractice Claims for Diagnostic Errors 1986-2010: An Analysis From the National Practitioner Data Bank,” BMJ Qual. Safety, 22(8), pp. 672–680. 10.1136/bmjqs-2012-001550 23610443

[10] Balogh, E. P., Miller, B. T., and Ball, J. R., eds., 2015, Improving Diagnosis in Health Care, The National Academies of Sciences, Engineering, and Medicine, Washington, DC.

[11] Singh, H., Meyer, A. N. D., and Thomas, E. J., 2014, “The Frequency of Diagnostic Errors in Outpatient Care: Estimations From Three Large Observational Studies Involving US Adult Populations,” BMJ Qual. Safety, 23(9), pp. 727–731. 10.1136/bmjqs-2013-002627 PMC4145460 24742777

[12] Newman-Toker, D. E., Nassery, N., Schaffer, A. C., Yu-Moe, C. W., Clemens, G. D., Wang, Z., Zhu, Y., ., 2024, “Burden of Serious Harms From Diagnostic Error in the USA,” BMJ Qual. Saf., 33(2), pp. 109–120. 10.1136/bmjqs-2021-014130 PMC10792094 37460118

[13] Watari, T., Tokuda, Y., Mitsuhashi, S., Otuki, K., Kono, K., Nagai, N., Onigata, K., and Kanda, H., 2020, “Factors and Impact of Physicians' Diagnostic Errors in Malpractice Claims in Japan,” PLoS One, 15(8), p. e0237145. 10.1371/journal.pone.0237145 32745150 PMC7398551

[14] Groome, D., and Eysenck, M., 2016, An Introduction to Applied Cognitive Psychology, Psychology Press, New York. 10.4324/9781315732954

[15] Topol, E., 2019, Deep Medicine: How Artificial Intelligence Can Make Healthcare Human Again, Hachette, London, UK.https://www.hachettebookgroup.com/titles/eric-topol/deep-medicine/9781541644632/

[16] Tversky, A., and Kahneman, D., 1974, “Judgment Under Uncertainty: Heuristics and Biases,” Science, 185(4157), pp. 1124–1131. 10.1126/science.185.4157.1124 17835457

[17] Coussens, S., 2018, “Behaving Discretely: Heuristic Thinking in the Emergency Department,” *Diagnosis: Interpreting the Shadows,* M. J. Balboni and J. R. Peteet eds., MIT Press, Cambridge, MA, pp. 183–204. 10.2139/ssrn.3743423

[18] Nataraj, C., 2021, “Can Nonlinear Dynamics Improve Medical Diagnostics?,” International Nonlinear Dynamics Conference (NODYCON) Keynote Speech, YouTube, San Bruno, CA, accessed Nov. 1, 2025, https://www.youtube.com/watch?v=z_wHNo5VXGU

[19] Singer, W., 2007, “Understanding the Brain. How Can Our Intuition Fail so Fundamentally When It Comes to Studying the Organ to Which It Owes Its Existence?,” EMBO Rep., 8(S1), p. S16. 10.1038/sj.embor.7400994 17726436 PMC3327521

[20] Newman, M. E. J., 2011, “Resource Letter CS-1: Complex Systems,” Am. J. Phys., 79(8), pp. 800–810. 10.1119/1.3590372

[21] Krakauer, D., 2022, “What is Complex System Science?,” Santa Fe Institute, Santa Fe, NM, accessed Nov. 1, 2025, https://www.santafe.edu/what-is-complex-systems-science

[22] Winfree, A. T., 1980, The Geometry of Biological Time, Vol. 8, Springer Biomathematics, New York.https://link.springer.com/book/10.1007/978-1-4757-3484-3

[23] Mackey, M. C., and Glass, L., 1977, “Oscillation and Chaos in Physiological Control Systems,” Science, 197(4300), pp. 287–289. 10.1126/science.267326 267326

[24] Glass, L., and Mackey, M. C., 1988, From Clocks to Chaos: The Rhythms of Life, Princeton University Press, Princeton, NJ.

[25] Goldberger, A. L., 1996, “Non-Linear Dynamics for Clinicians: Chaos Theory, Fractals, and Complexity at the Bedside,” Lancet, 347(9011), pp. 1312–1314. 10.1016/S0140-6736(96)90948-4 8622511

[26] Glass, L., 2001, “Synchronization and Rhythmic Processes in Physiology,” Nature, 410(6825), pp. 277–284. 10.1038/35065745 11258383

[27] Murray, J. D., 2002, Mathematical Biology I: An Introduction (Interdisciplinary Applied Mathematics), 3rd ed., Vol. 17, Springer, New York.https://link.springer.com/book/10.1007/b98868

[28] Noble, D., 2006, The Music of Life: Biology Beyond Genes, Oxford University Press, Oxford, UK.

[29] Kantz, H., and Schreiber, T., 2004, “Nonlinear Time Series Analysis,” Chaos, 14(1), pp. 501–506. 10.1063/1.1648864

[30] Goldberger, A. L., Amaral, L. A. N., Hausdorff, J. M., Ivanov, P. C., Peng, C.-K., and Stanley, H. E., 2002, “Fractal Dynamics in Physiology: Alterations With Disease and Aging,” Proc. Natl. Acad. Sci., 99(suppl_1), pp. 2466–2472. 10.1073/pnas.012579499 11875196 PMC128562

[31] Lainscsek, C., Cash, S. S., Sejnowski, T. J., and Kurths, J., 2021, “Dynamical Ergodicity DDA Reveals Causal Structure in Time Series,” Chaos, 31(10), p. 103108. 10.1063/5.0063724 34717330 PMC12640249

[32] Cheffer, A., Savi, M. A., Pereira, T. L., and de Paula, A. S., 2021, “Heart Rhythm Analysis Using a Nonlinear Dynamics Perspective,” Appl. Math. Modell., 96, pp. 152–176. 10.1016/j.apm.2021.03.014

[33] Nagamori, A., Laine, C. M., Loeb, G. E., and Valero-Cuevas, F. J., 2021, “Force Variability is Mostly Not Motor Noise: Theoretical Implications for Motor Control,” PLoS Comput. Biol., 17(3), p. e1008707. 10.1371/journal.pcbi.1008707 33684099 PMC7971898

[34] Mezentseva, L. V., 2021, “The Features of the Nonlinear Dynamics of the Microcirculation Parameters of the Upper Limbs Under Perturbation,” Biophysics, 66(1), pp. 149–154. 10.1134/S0006350921010206

[35] Bélair, J., Nekka, F., and Milton, J. G., 2021, “Introduction to Focus Issue: Dynamical Disease: A Translational Approach,” Chaos, 31(6), p. 060401. 10.1063/5.0058345 34241319

[36] Gois, S. R., and Savi, M. A., 2009, “An Analysis of Heart Rhythm Dynamics Using a Three-Coupled Oscillator Model,” Chaos, Solitons Fractals, 41(5), pp. 2553–2565. 10.1016/j.chaos.2008.09.040

[37] Ferreira, B. B., de Paula, A. S., and Savi, M. A., 2011, “Chaos Control Applied to Heart Rhythm Dynamics,” Chaos, Solitons Fractals, 44(8), pp. 587–599. 10.1016/j.chaos.2011.05.009

[38] Babloyantz, A., and Destexhe, A., 1986, “Low-Dimensional Chaos in an Instance of Epilepsy,” Proc. Natl. Acad. Sci., 83(10), pp. 3513–3517. 10.1073/pnas.83.10.3513 3085091 PMC323547

[39] Stam, C. J., 2005, “Nonlinear Dynamical Analysis of EEG and MEG: Review of an Emerging Field,” Clin. Neurophysiol., 116(10), pp. 2266–2301. 10.1016/j.clinph.2005.06.011 16115797

[40] Lehnertz, K., and Elger, C. E., 2000, “Spatio-Temporal Dynamics of the Primary Epileptogenic Area in Temporal Lobe Epilepsy Characterized by Neuronal Complexity Loss,” Phys. D Nonlinear Phenom., 140(3–4), pp. 358–369. 10.1016/0013-4694(95)00071-6 7649002

[41] Andrzejak, R. G., Lehnertz, K., Mormann, F., Rieke, C., David, P., and Elger, C. E., 2001, “Indications of Nonlinear Deterministic and Finite-Dimensional Structures in Time Series of Brain Electrical Activity: Dependence on Recording Region and Brain State,” Phys. Rev. E Stat., Nonlinear, Soft Matter Phys., 64(6 Pt 1), p. 061907. 10.1103/PhysRevE.64.061907 11736210

[42] Mormann, F., Kreuz, T., Andrzejak, R. G., David, P., Lehnertz, K., and Elger, C. E., 2003, “Epileptic Seizures Are Preceded by a Decrease in Synchronization,” Epilepsy Res., 53(3), pp. 173–185. 10.1016/S0920-1211(03)00002-0 12694925

[43] Glass, L., 1991, “Cardiac Arrhythmias and Circle Maps—A Classical Example of Nonlinear Dynamics,” Chaos, 1(1), pp. 13–19. 10.1063/1.165810 12779891

[44] Mäkikallio, T. H., Koistinen, J., Jordaens, L., Tulppo, M. P., Wood, N., Golosarsky, B., Peng, C.-K., Goldberger, A. L., and Huikuri, H. V., 1999, “Heart Rate Dynamics Before Spontaneous Onset of Ventricular Fibrillation in Patients with Healed Myocardial Infarcts,” Am. J. Cardiol., 83(6), pp. 880–884. 10.1016/s0002-9149(98)01068-6 10190403

[45] Lipsitz, L. A., and Goldberger, A. L., 1992, “Loss of “Complexity” and Aging: Potential Applications of Fractals and Chaos Theory to Senescence,” JAMA, 267(13), pp. 1806–1809. 10.1001/jama.1992.03480130122036 1482430

[46] Lipsitz, L. A., 2004, “Physiological Complexity, Aging, and the Path to Frailty,” Sci. Aging Knowl. Environ., 2004(16), p. pe16. 10.1126/sageke.2004.16.pe16 15103055

[47] Sharma, R., Sharma, D., and Singh, V., 2009, “Deterministic Chaos and Fractal Complexity in the Heart,” Open Cardiovasc. Med. J., 3(1), pp. 110–123. 10.2174/1874192400903010110 19812706 PMC2757669

[48] Torre, K., Balasubramaniam, E., and Bardy, B., 2019, “Fractal Properties in Sensorimotor Variability Unveil Internal Adaptations,” Sci. Rep., 9(1), p. 15736. 10.1038/s41598-019-52091-y 31673034 PMC6823488

[49] Ozel, C., Demir, H. H., Sari, E. A., and Bas, Y. C., 2023, “A Simple Approach to Determine Loss of Physiological Complexity,” Chaos, Solitons Fractals, 172, p. 113532. 10.1016/j.chaos.2023.113832

[50] Takens, F., 1981, “Detecting Strange Attractors in Turbulence,” Dynamical Systems and Turbulence, Warwick 1980 (Lecture Notes in Mathematics), Vol. 898, Springer‐Verlag, Berlin, pp. 366–381.

[51] Sauer, T., Yorke, J. A., and Casdagli, M., 1991, “Embedology,” J. Stat. Phys., 65(3–4), pp. 579–616. 10.1007/BF01053745

[52] Oseledec, V. I., 1968, “A Multiplicative Ergodic Theorem: Lyapunov Characteristic Numbers for Dynamical Systems,” Trans. Moscow Math. Soc., 19, pp. 197–231.

[53] Wolf, A., Swift, J. B., Swinney, H. L., and Vastano, J. A., 1985, “Determining Lyapunov Exponents From a Time Series,” Phys. D Nonlinear Phenom., 16(3), pp. 285–317. 10.1016/0167-2789(85)90011-9

[54] Ivanov, P. C., Amaral, L. A. N., Goldberger, A. L., Havlin, S., Rosenblum, M. G., Stanley, H. E., and Struzik, Z. R., 1999, “Multifractality in Human Heartbeat Dynamics,” Nature, 399(6735), pp. 461–465. 10.1038/20924 10365957

[55] Magin, R. L., 2010, “Fractional Calculus Models of Complex Dynamics in Biological Tissues,” Comput. Math. Appl., 59(5), pp. 1586–1593. 10.1016/j.camwa.2009.08.039

[56] West, B. J., 2014, “A Mathematics for Medicine: The Network Effect,” Front. Physiol., 5, p. 456. 10.3389/fphys.2014.00456 25538622 PMC4260484

[57] Vinet, A., Chialvo, D. R., Michaels, D. C., and Jalife, J., 1990, “Nonlinear Dynamics of Rate-Dependent Activation in Models of Single Cardiac Cells,” Circ. Res., 67(6), pp. 1510–1524. 10.1161/01.RES.67.6.1510 2245510

[58] Romero, L., Alvarez-Lacalle, E., and Shiferaw, Y., 2019, “Stochastic Coupled Map Model of Subcellular Calcium Cycling in Cardiac Cells,” Chaos, 29(2), p. 023125. 10.1063/1.5063462 30823735 PMC7043839

[59] Takembo, C. N., Mvogo, A., Fouda, H. P. E., and Kofane, T. C., 2019, “Localized Modulated Wave Solution of Diffusive FitzHugh-Nagumo Cardiac Networks Under Magnetic Flow Effect,” Nonlinear Dyn., 95(2), pp. 1079–1098. 10.1007/s11071-018-4617-z

[60] Guevara, M. R., Glass, L., and Shrier, A., 1981, “Phase Locking, Period-Doubling Bifurcations, and Irregular Dynamics in Periodically Stimulated Cardiac Cells,” Science, 214(4527), pp. 1350–1353. 10.1126/science.7313693 7313693

[61] Glass, L., 1991, “Alternans and Period-Doubling Bifurcations in Cardiac Cells,” Ann. New York Acad. Sci., 591(1), pp. 316–327.

[62] Frank, O., 1899, “Die Grundform Des Arterielen Pulses Erste Abhandlung: Mathematische Analyse,” Z. Biol., 37, pp. 483–526.

[63] Westerhof, N., Lankhaar, J.-W., and Westerhof, B. E., 2009, “The Arterial Windkessel,” Med. Biol. Eng. Comput., 47(2), pp. 131–141. 10.1007/s11517-008-0359-2 18543011

[64] Parker, K. H., 2009, “A Brief History of Arterial Wave Mechanics,” Med. Biol. Eng. Comput., 47(2), pp. 111–118. 10.1007/s11517-009-0440-5 19198914 PMC2644374

[65] Aboelkassem, Y., Virag, Z., and Virag, V., 2019, “A Hybrid Windkessel–Womersley Model for Blood Flow in Arterial System,” J. Theor. Biol., 462, pp. 499–513. 10.1016/j.jtbi.2018.12.005 30528559

[66] Bahloul, M. A., Aboelkassem, Y., Belkhatir, Z., and Laleg-Kirati, T. M., 2023, “Fractional-Order Modified Windkessel Model of the Human Arterial Vascular System,” IEEE Biomedical Circuits and Systems Conference (BioCAS), Toronto, ON, Canada, Oct. 19–21, pp. 1–5. 10.1109/BioCAS58349.2023.10388708

[67] Fåhræus, R., and Lindqvist, T., 1931, “The Viscosity of the Blood in Narrow Capillary Tubes,” Am. J. Physiol., 96(3), pp. 562–568. 10.1152/ajplegacy.1931.96.3.562

[68] Fåhræus, R., and Lindqvist, T., 1931, “Die Viskosität Des Blutes in Engen Kapillaren,” Klin. Wochensch., 10(32), pp. 1688–1690.

[69] Pries, A. R., Secomb, T. W., and Gaehtgens, P., 1996, “Biophysical Aspects of Blood Flow in the Microvasculature,” Cardiovasc. Res., 32(4), pp. 654–667. 10.1016/S0008-6363(96)00065-X 8915184

[70] Ascolese, M., Farina, A., and Fasano, A., 2019, “The Fåhræus–Lindqvist Effect in Small Blood Vessels,” J. Biol. Phys., 45, pp. 379–394. 10.1007/s10867-019-09534-4 31792778 PMC6917688

[71] Chebbi, R., 2015, “Modeling of the Fåhræus–Lindqvist Effect,” J. Biol. Phys., 41(3), pp. 313–326. 10.1007/s10867-015-9376-1 25702195 PMC4456490

[72] Stergiou, Y. G., Keramydas, A. T., Anastasiou, A. D., Mouza, A. A., and Paras, S. V., 2019, “Experimental and Numerical Study of Blood Flow in μ-Vessels: Influence of the Fåhræus–Lindqvist Effect,” Fluids, 4(3), p. 143. 10.3390/fluids4030143

[73] Baeyens, N., and Schwartz, M. A., 2016, “Biochemical and Biomechanical Signaling in Endothelial Cells,” Nat. Rev. Mol. Cell Biol., 17(10), pp. 611–622.27461391

[74] Chiu, J.-J., and Chien, S., 2011, “Effects of Disturbed Flow on Vascular Endothelium: Pathophysiological Basis and Clinical Perspectives,” Physiol. Rev., 91(1), pp. 327–387. 10.1152/physrev.00047.2009 21248169 PMC3844671

[75] Cheng, H., Zhong, W., Wang, L., Zhang, Q., Ma, X., Wang, Y., Wang, S., ., 2023, “Effects of Shear Stress on Vascular Endothelial Functions in Health and Disease,” Life Sci., 330, p. 121852.

[76] Cheng, C. K., Wang, N., Wang, L., and Huang, Y., 2025, “Biophysical and Biochemical Roles of Shear Stress on Endothelial Cells,” Circulation Research, 136(7), 752–772. 10.1161/CIRCRESAHA.124.325685 40146803 PMC11949231

[77] Zhou, M., Yu, Y., Chen, R., Liu, X., Hu, Y., Ma, Z., Gao, L., Jian, W., and Wang, L., 2023, “Wall Shear Stress and Its Role in Atherosclerosis,” Front. Cardiovasc. Med., 10, p. 1083547. 10.3389/fcvm.2023.1083547 37077735 PMC10106633

[78] Luisi, C. A., Witter, T. L., Nikoubashman, O., Wiesmann, M., Steinseifer, U., and Neidlin, M., 2024, “Evaluating the Accuracy of Cerebrovascular Computational Fluid Dynamics Modeling Through Time-Resolved Experimental Validation,” Sci. Rep., 14(1), p. 8194. 10.1038/s41598-024-58925-8 38589554 PMC11001858

[79] Hu, M., Wang, S., Peng, Z., Chen, W., Li, Z., and Zhang, T., 2025, “Computational Fluid Dynamics Modelling of Hemodynamics in Aortic Aneurysm and Dissection: A Review,” Front. Bioeng. Biotechnol., 13, p. 1556091. 10.3389/fbioe.2025.1556091 40190707 PMC11968685

[80] Tunedal, K., Ebbers, T., and Cedersund, G., 2025, “Uncertainty in Cardiovascular Digital Twins Despite Non-Normal Errors in 4D Flow MRI: Identifying Reliable Biomarkers Such as Ventricular Relaxation Rate,” Comput. Biol. Med., 188, p. 109878. 10.1016/j.compbiomed.2025.109878 39987701

[81] Sel, K., Hawkins-Daarud, A., Chaudhuri, A., Osman, D., Bahai, A., Paydarfar, D., Willcox, K., Chung, C., and Jafari, R., 2025, “Survey and Perspective on Verification, Validation, and Uncertainty Quantification of Digital Twins for Precision Medicine,” NPJ Dig. Med., 8(1), p. 40. 10.1038/s41746-025-01447-y PMC11742391 39825103

[82] Task Force of the European Society of Cardiology and the North American Society of Pacing and Electrophysiology, 1996, “Heart Rate Variability: Standards of Measurement, Physiological Interpretation and Clinical Use,” Eur. Heart J., 17(3), pp. 354–381. 10.1093/oxfordjournals.eurheartj.a014868 8737210

[83] Peng, C. K., Havlin, S., Stanley, H. E., and Goldberger, A. L., 1995, “Quantification of Scaling Exponents and Crossover Phenomena in Nonstationary Heartbeat Time Series,” Chaos (Woodbury, NY), 5(1), pp. 82–87. 10.1063/1.166141 11538314

[84] Lin, D. C., and Sharif, A., 2010, “Common Multifractality in the Heart Rate Variability and Brain Activity of Healthy Humans”, Chaos, 20(2), p. 023121. 10.1063/1.3427639 20590317

[85] Costa, M., Goldberger, A. L., and Peng, C.-K., 2002, “Multiscale Entropy Analysis of Complex Physiologic Time Series,” Phys. Rev. Lett., 89(6), p. 068102. 10.1103/PhysRevLett.89.068102 12190613

[86] Lindén, M., Nayak, S. K., Bit, A., Dey, A., Mohapatra, B., and Pal, K., 2018, “A Review on the Nonlinear Dynamical System Analysis of Electrocardiogram Signal,” J. Healthcare Eng., 2018, p. 6920420. 10.1155/2018/6920420 PMC5954865 29854361

[87] Henriques, T., Ribeiro, M., Teixeira, A., Castro, L., Antunes, L., and Costa-Santos, C., 2020, “Nonlinear Methods Most Applied to Heart-Rate Time Series: A Review,” Entropy, **22**(3), p. 309. 10.3390/e22030309 33286083 PMC7516766

[88] Quiroz-Juárez, M., Jiménez-Ramírez, O., Vázquez-Medina, R., Breña-Medina, V., Aragón, J., and Barrio, R., 2019, “Generation of ECG Signals From a Reaction-Diffusion Model Spatially Discretized,” Sci. Rep., 9(1), p. 19000. 10.1038/s41598-019-55448-5 31831864 PMC6908715

[89] Nayak, S. K., Bit, A., Dey, A., Mohapatra, B., and Pal, K., 2018, “A Review on the Nonlinear Dynamical System Analysis of Electrocardiogram Signal,” J. Healthcare Eng., 2018, pp. 1–19. 10.1155/2018/6920420 PMC5954865 29854361

[90] Lopes, F. R., and Morgado de Gois, J. A., 2018, “ECG Model Parameters Optimization and Space State Reconstruction,” J. Braz. Soc. Mech. Sci. Eng., 40(8), p. 399. 10.1007/s40430-018-1313-3

[91] Ladeira, G., Lima, G. V., Ribeiro, M. A., and Balthazar, J. M., 2021, “Kaplan Oscillator Configuration by Means of Bifurcation Diagrams to Generate the P and T Waves, in Addition QRS Complex of the ECG,” Braz. J. Phys., 51(3), pp. 644–652. 10.1007/s13538-021-00898-4

[92] Das, S., and Maharatna, K., 2013, “Fractional Dynamical Model for the Generation of ECG Like Signals From Filtered Coupled Van-Der Pol Oscillators,” Comput. Methods Prog. Biomed., 112(3), pp. 490–507. 10.1016/j.cmpb.2013.08.012 24028797

[93] Gul, M. U., Masood, B., Khalil, H., and Nazar, W., 2017, “Modeling & Simulation of Nonlinear Dynamics of Periodic Cardiac Pacemaker Using Bond Graph Techniques,” 3rd International Conference on Engineering Technologies and Social Sciences (ICETSS), Bangkok, Thailand, Aug. 7–8, pp. 1–6. 10.1109/ICETSS.2017.8324182

[94] Cheffer, A., Ritto, T. G., and Savi, M. A., 2021, “Uncertainty Analysis of Heart Dynamics Using Random Matrix Theory,” Int. J. Non-Linear Mech., 129, p. 103653. 10.1016/j.ijnonlinmec.2020.103653

[95] Brignol, A., and Al-Ani, T., 2014, “A Phase-Space Based Algorithm for Detecting Different Types of Artefacts,” XIII Mediterranean Conference on Medical and Biological Engineering and Computing (MEDICON 2013), Springer, Seville, Spain, Sep. 25–28, pp. 599–602. 10.1007/978-3-319-00846-2_149

[96] Varanis, M., Hemmati, S., Filipus, M. C., Abreu, F. L. D., Balthazar, J. M., and Nataraj, C., 2024, “An Overview on Time-Frequency Effects of ECG Signals Using Synchroextracting Transform,” Advances in Nonlinear Dynamics, W. Lacarbonara, ed., Vol. III, Springer Nature Switzerland, Cham, Switzerland, pp. 591–600.

[97] Tang, S.-Y., Ma, H.-P., Hung, C.-S., Kuo, P.-H., Lin, C., Lo, M.-T., Hsu, H.-H., ., 2021, “The Value of Heart Rhythm Complexity in Identifying High-Risk Pulmonary Hypertension Patients,” Entropy, 23(6), p. 753. 10.3390/e23060753 34203737 PMC8232109

[98] Tsai, C.-H., Ma, H.-P., Lin, Y.-T., Hung, C.-S., Huang, S.-H., Chuang, B.-L., Lin, C., Lo, M.-T., Peng, C.-K., and Lin, Y.-H., 2020, “Usefulness of Heart Rhythm Complexity in Heart Failure Detection and Diagnosis,” Sci. Rep., 10(1), p. 14916. 10.1038/s41598-020-71909-8 32913306 PMC7483411

[99] Tsai, C., Huang, J., Lin, C., Ma, H., Lo, M., Liu, L. D., Lin, L., ., 2020, “Heart Rhythm Complexity Predicts Long‐Term Cardiovascular Outcomes in Peritoneal Dialysis Patients: A Prospective Cohort Study,” J. Am. Heart Assoc., 9(2), p. e013036. 10.1161/JAHA.119.013036 31910780 PMC7033842

[100] Cui, X., Tian, L., Li, Z., Ren, Z., Zha, K., Wei, X., and Peng, C.-K., 2020, “On the Variability of Heart Rate Variability–Evidence From Prospective Study of Healthy Young College Students,” Entropy, 22(11), p. 1302. 10.3390/e22111302 33263356 PMC7711844

[101] Mao, X., Shang, P., Yang, A. C., and Peng, C.-K., 2020, “Multiscale Cumulative Residual Distribution Entropy and Its Applications on Heart Rate Time Series,” Nonlinear Dyn., 101(4), pp. 2357–2368. 10.1007/s11071-020-05934-7

[102] Veerabhadrappa, R., Hettiarachchi, I. T., and Bhatti, A., 2021, “Using Recurrence Quantification Analysis to Quantify the Physiological Synchrony in Dyadic ECG Data,” IEEE International Systems Conference (SysCon), Vancouver, BC, Canada, Apr. 12–15, pp. 1–8. 10.1109/SysCon48628.2021.9447089

[103] Drzewiecki, G., and Drzewiecki, G., 2021, “Topics in Nonlinear Dynamics,” Fundamentals of Chaos and Fractals for Cardiology, Springer International Publishing, Cham, Switzerland, pp. 9–19.

[104] Biasci, V., Sacconi, L., Cytrynbaum, E. N., Pijnappels, D. A., De Coster, T., Shrier, A., Glass, L., and Bub, G., 2020, “Universal Mechanisms for Self-Termination of Rapid Cardiac Rhythm,” Chaos, 30(12), p. 121107. 10.1063/5.0033813 33380016

[105] Alonso-Atienza, F., Morgado, E., Fernandez-Martinez, L., Garcia-Alberola, A., and Rojo-Alvarez, J. L., 2014, “Detection of Life-Threatening Arrhythmias Using Feature Selection and Support Vector Machines,” IEEE Trans. Biomed. Eng., 61(3), pp. 832–840. 10.1109/TBME.2013.2290800 24239968

[106] Ksela, J., Avbelj, V., and Kalisnik, J. M., 2015, “Multifractality in Heartbeat Dynamics in Patients Undergoing Beating-Heart Myocardial Revascularization,” Comput. Biol. Med., 60, pp. 66–73. 10.1016/j.compbiomed.2015.02.012 25756703

[107] Wisdom, K. M., Delp, S. L., and Kuhl, E., 2015, “Use It or Lose It: Multiscale Skeletal Muscle Adaptation to Mechanical Stimuli,” Biomech. Model. Mechanobiol., 14(2), pp. 195–215. 10.1007/s10237-014-0607-3 25199941 PMC4352121

[108] Lieber, R. L., 2010, Skeletal Muscle Structure, Function, and Plasticity: The Physiological Basis of Rehabilitation, 3rd ed., Lippincott Williams & Wilkins, Philadelphia, PA.https://catalog.nlm.nih.gov/discovery/fulldisplay?docid=alma9915244803406676&context=L&vid=01NLM_INST:01NLM_INST&lang=en&search_scope=CATONLY&adaptor=Local%20Search%20Engine&tab=CATONLY&query=creator,equals,Lieber,%20Richard%20L.,AND&mode=advanced&offset=0

[109] Hill, A. V., 1938, “The Heat of Shortening and the Dynamic Constants of Muscle,” Proc. R. Soc. London. Ser. B Biol. Sci., 126(843), pp. 136–195. 10.1098/rspb.1938.0050

[110] Tsianos, G. A., and Loeb, G. E., 2015, “Physiology and Computational Principles of Muscle Force Generation,” Encyclopedia of Computational Neuroscience, D. Jaeger and R. Jung, eds., Springer, New York, pp. 2379–2395.

[111] Zhang, X., Chan, F. K., Parthasarathy, T., and Gazzola, M., 2019, “Modeling and Simulation of Complex Dynamic Musculoskeletal Architectures,” Nat. Commun., 10(1), p. 4825. 10.1038/s41467-019-12759-5 31645555 PMC6811595

[112] González, A., Cerda-Lugo, A., Cardenas, A., Maya, M., and Piovesan, D., 2019, “A Third-Order Model of Hip and Ankle Joints During Balance Recovery: Modeling and Parameter Estimation,” ASME J. Comput. Nonlinear Dyn., 14(10), p. 101001. 10.1115/1.4042527

[113] Trkov, M., Chen, K., and Yi, J., 2019, “Bipedal Model and Hybrid Zero Dynamics of Human Walking With Foot Slip,” ASME J. Comput. Nonlinear Dyn., 14(10), p. 101002. 10.1115/1.4043360

[114] Chumacero-Polanco, E., Yang, J., and Chagdes, J., 2019, “Numerical Nonlinear Analysis for Dynamic Stability of an Ankle-Hip Model of Balance on a Balance Board,” ASME J. Comput. Nonlinear Dyn., 14(10), p. 101003. 10.1115/1.4042693

[115] Hausdorff, J. M., Peng, C.-K., Ladin, Z., Wei, J. Y., and Goldberger, A. L., 1996, “Is Walking a Random Walk? Evidence for Long-Range Correlations in Stride Interval of Human Gait,” Proc. Natl. Acad. Sci., 93(2), pp. 702–706. 10.1152/jappl.1995.78.1.349 7713836

[116] West, B. J., and Scafetta, N., 2003, “Nonlinear Dynamical Model of Human Gait,” PRE, 67(5 Pt 1), p. 051917. 10.1103/PhysRevE.67.051917 12786188

[117] Hausdorff, J. M., 2001, “Gait Variability: Methods, Modeling and Meaning,” J. Appl. Physiol., 92(1), pp. 557–565. 10.1186/1743-0003-2-19 PMC1185560 16033650

[118] Stergiou, N., and Decker, L. M., 2011, “Human Movement Variability, Nonlinear Dynamics, and Pathology: Is There a Connection?,” Human Movement Sci., 30(5), pp. 869–888. 10.1016/j.humov.2011.06.002 PMC3183280 21802756

[119] Su, D., Liu, Z., Yang, S., Wang, Y., Ma, H., Manor, B., Hausdorff, J. M., ., 2021, “O-MD003. The Complexity of Involuntary Hand Motion Distinguishes Between Essential and Parkinsonian Tremor,” Clin. Neurophysiol., 132(8), p. e72. 10.1016/j.clinph.2021.02.142 34148777

[120] Vaillancourt, D. E., and Newell, K. M., 2000, “The Dynamics of Resting and Postural Tremor in Parkinson's Disease,” Biol. Cybern., 82(4), pp. 269–280. 11068241 10.1016/s1388-2457(00)00467-3

[121] Beuter, A., and Vasilakos, K., 1995, “Tremor: Analysis of Complexity and Filtering of Physiological Signals,” Chaos, 5(1), pp. 56–60.

[122] Vaillancourt, D. E., Slifkin, A. B., and Newell, K. M., 2001, “Intermittency in the Visual Control of Force in Parkinson's Disease,” NeuroImage, 14(4), pp. 702–710. 10.1007/s002210100699 11374078

[123] Sturman, M. M., Vaillancourt, D. E., and Corcos, D. M., 2005, “Effects of Aging and Parkinson's Disease on Motor Variability,” Move. Disorders, 20(12), pp. 1334–1341.

[124] Rissanen, S. M., Koivu, M., Hartikainen, P., and Pekkonen, E., 2021, “Ambulatory Surface Electromyography With Accelerometry for Evaluating Daily Motor Fluctuations in Parkinson's Disease,” Clin. Neurophysiol., 132(2), pp. 469–479. 10.1016/j.clinph.2020.11.039 33450567

[125] Little, M. A., McSharry, P. E., Hunter, E. J., Spielman, J., and Ramig, L. O., 2012, “Suitability of Dysphonia Measurements for Telemonitoring of Parkinson's Disease,” Front. Neurol., 3, p. 92. 21399744 10.1109/TBME.2008.2005954 PMC3051371

[126] Badicu, G., Clemente, F. M., and Murawska-Cialowicz, E., 2022, “Biological Mechanisms Underlying Physical Fitness and Sports Performance: An Editorial,” Biology, **11**(10), p. 1542. 10.3390/biology11101425 PMC9598305 36290329

[127] Levack-Payne, W., 2022, “Mechanistic Evidence and Exercise Interventions: Causal Claims, Extrapolation, and Implementation,” J. Eval. Clin. Pract., 28(5), pp. 745–751. 10.1111/jep.13748 35971196 PMC9804705

[128] Prodromos, C. C., Han, Y., Rogowski, J., Joyce, B., and Shi, K., 2007, “A Meta-Analysis of the Incidence of Anterior Cruciate Ligament Tears as a Function of Gender, Sport, and a Knee Injury-Reduction Regimen,” Arthroscopy, 23(12), pp. 1320–1325. 10.1016/j.arthro.2007.07.003 18063176

[129] Waldén, M., Hägglund, M., Werner, J., and Ekstrand, J., 2011, “The Epidemiology of Anterior Cruciate Ligament Injury in Football (Soccer): A Review of the Literature From a Gender-Related Perspective,” Knee Surg., Sports Traumatology, Arthroscopy: J. ESSKA, 19(1), pp. 3–10. 10.1007/s00167-010-1172-7 20532868

[130] Balagué, N., Hristovski, R., Almarcha, M. D. C., Garcia-Retortillo, S., and Ivanov, P., 2020, “Network Physiology of Exercise: Vision and Perspectives,” Front. Physiol., 11, p. 568. 10.3389/fphys.2020.611550 PMC7759565 33362584

[131] Sorensen, T. J., 1978, “A Physiological Model of Glucose Metabolism in Man and Its Use to Design and Assess Improved Insulin Therapies for Diabetes,” Ph.D. thesis, University of California, Berkeley, CA.

[132] Panunzi, S., Pompa, M., Borri, A., Piemonte, V., and De Gaetano, A., 2020, “A Revised Sorensen Model: Simulating Glycemic and Insulinemic Response to Oral and Intra-Venous Glucose Load,” PLoS One, 15(8), p. e0237215. 10.1371/journal.pone.0237215 32797106 PMC7428140

[133] Goodwin, B. C., 1965, “Oscillatory Behavior in Enzymatic Control Processes,” Adv. Enzyme Regul., 3, pp. 425–437. 10.1016/0065-2571(65)90067-1 5861813

[134] Sturis, J., Polonsky, K. S., Mosekilde, E., and Van Cauter, E., 1991, “Computer Model for Mechanisms Underlying Ultradian Oscillations of Insulin and Glucose,” Diabetes, 40(2), pp. 150–154. 2035636 10.1152/ajpendo.1991.260.5.E801

[135] Guyton, A. C., Coleman, T. G., Cowley, A. V. J., Scheel, K. W., Manning, R. D. J., and Norman, R. A. J., 1972, “Arterial Pressure Regulation. Overriding Dominance of the Kidneys in Long-Term Regulation and in Hypertension,” Am. J. Med., 52(5), pp. 584–594. 10.1016/0002-9343(72)90050-2 4337474

[136] Hovorka, R., Canonico, V., Chassin, L. J., Haueter, U., Massi-Benedetti, M., Federici, M. O., Pieber, T. R., ., 2004, “Nonlinear Model Predictive Control of Glucose Concentration in Subjects With Type 1 Diabetes,” Physiol. Meas., 25(4), pp. 905–920. 10.1088/0967-3334/25/4/010 15382830

[137] Xu, J., and Pei, L., 2010, “Effects of Technological Delay on Insulin and Blood Glucose in a Physiological Model,” Int. J. Non-Linear Mech., 45(6), pp. 628–633. 10.1016/j.ijnonlinmec.2010.03.006

[138] Topp, B., Promislow, K., de Vries, G., Miura, R. M., and Finegood, D. T., 2000, “A Model of Beta-Cell Mass, Insulin, Glucose, and Receptor Dynamics With Applications to Diabetes,” J. Theor. Biol., 206, pp. 605–619. 10.1006/jtbi.2000.2150 11013117

[139] Dalla Man, C., Micheletto, F., Lv, D., Breton, M., Kovatchev, B., and Cobelli, C., 2014, “The UVA/PADOVA Type 1 Diabetes Simulator: New Features,” J. Diabetes Sci. Technol., 8(1), pp. 26–34. 10.1177/1932296813514502 24876534 PMC4454102

[140] Ramanujan, V. K., and Herman, B. A., 2007, “Aging Process Modulates Nonlinear Dynamics in Liver Cell Metabolism*,” J. Biol. Chem., 282(26), pp. 19217–19226. 10.1074/jbc.M700572200 17446172

[141] Hatzimanikatis, V., 1999, “Nonlinear Metabolic Control Analysis,” Metab. Eng., 1(1), pp. 75–87. 10.1006/mben.1998.0108 10935756

[142] Keener, J., and Sneyd, J., 2009, Mathematical Physiology (Interdisciplinary Applied Mathematics), 2nd ed., Vol. 8, Springer, New York.

[143] Voit, E. O., 2000, Computational Analysis of Biochemical Systems: A Practical Guide for Biochemists and Molecular Biologists, Cambridge University Press, Cambridge, UK.

[144] Carvalho, A. R., and Zin, W. A., 2011, “Respiratory System Dynamical Mechanical Properties: Modeling in Time and Frequency Domain,” Biophys. Rev., 3(2), pp. 71–84. 10.1007/s12551-011-0048-5 28510005 PMC5418399

[145] Thamrin, C., Frey, U., Kaminsky, D. A., Reddel, H. K., Seely, A. J. E., Suki, B., and Sterk, P. J., 2016, “Systems Biology and Clinical Practice in Respiratory Medicine. The Twain Shall Meet,” Am. J. Resp. Crit. Care Med., 194(9), pp. 1053–1061. 10.1164/rccm.201511-2288PP 27556336 PMC5114447

[146] Suki, B., Barabási, A.-L., Hantos, Z., Peták, F., and Stanley, H. E., 1994, “Lung Tissue Viscoelasticity: A Structural Damping Model,” Nature, 368(6472), pp. 615–618. 10.1038/368615a0 8145846

[147] Bates, J. H. T., 2009, Lung Mechanics: An Inverse Modeling Approach, Cambridge University Press, Cambridge, UK.

[148] Bates, J. H. T., and Suki, B., 2014, “Lung Tissue Mechanics: Nonlinear, Multiscale, and Clinical Relevance,” Annu. Rev. Biomed. Eng., 16, pp. 77–102. 10.1146/annurev-bioeng-071813-10484324819476

[149] van Eijk, J. A., Doeleman, L. C., Loer, S. A., Koster, R. W., van Schuppen, H., and Schober, P., 2024, “Ventilation During Cardiopulmonary Resuscitation: A Narrative Review,” Resuscitation, 203, p. 110366. 10.1016/j.resuscitation.2024.110366 39181499

[150] Algahtani, A. I., colleagues, 2024, “Ventilation and Oxygenation During and After Adult CPR: A Review,” Resp. Care, 69(2), p. 12427. 10.4187/respcare.12427

[151] Morgan, R. W., Reeder, R. W., Bender, D., Cooper, K. K., Friess, S. H., Graham, K., Meert, K. L., ., 2023, “Associations Between End-Tidal Carbon Dioxide During Pediatric Cardiopulmonary Resuscitation, CPR Quality, and Survival,” Circulation, **149**(5), pp. 367–378. 10.1161/CIRCULATIONAHA.123.066659 37929615 PMC10841728

[152] Sahu, A. K., Behera, S. B., and Jindal, A. K., 2020, “Six-Dial Ventilator Strategy During Cardiopulmonary Resuscitation,” Indian J. Crit. Care Med., 24(8), pp. 671–674. 10.5005/jp-journals-10071-23464 PMC7435081 32863648

[153] Jalali, A., Simpao, A. F., Nadkarni, V. M., Berg, R. A., and Nataraj, C., 2017, “A Novel Nonlinear Mathematical Model of Thoracic Wall Mechanics During Cardiopulmonary Resuscitation Based on a Porcine Model of Cardiac Arrest,” J. Med. Syst., 41(2), p. 20. 10.1007/s10916-016-0676-1 27987159

[154] Orlob, S., Wittig, J., Hobisch, C., Auinger, D., Honnef, G., Fellinger, T., Ristl, R., ., 2021, “Reliability of Mechanical Ventilation During Continuous Chest Compressions: A Crossover Study in Human Cadavers,” Scand. J. Trauma, Resuscit. Emer. Med., 29(1), p. 148. 10.1186/s13049-021-00921-2 PMC8316711 34321068

[155] Gottschalk, A., Geitz, K. A., Richter, D. W., Ogilvie, M. D., and Pack, A. I., 1992, “Nonlinear Dynamics of a Model of the Central Respiratory Pattern Generator,” Control of Breathing and Its Modeling Perspective, Y. Honda, Y. Miyamoto, K. Konno, and J. G. Widdicombe, eds., Springer US, Boston, MA, pp. 51–55.

[156] Bruce, E. N., 1994, “Nonlinear Dynamics of Respiratory Reflexes,” IFAC Proc. Vols, 27(1), pp. 497–500. 10.1016/S1474-6670(17)46316-6

[157] Burioka, N., Suyama, H., Sako, T., Mitata, M., Takeshima, T., Endo, M., Kurai, J., ., 2002, “Non-Linear Dynamics Applied to Human Respiratory Movement During Sleep,” Biomed. Pharmacother., 56(Suppl 2), pp. 370–373. 10.1016/S0753-3322(02)00320-7 12653197

[158] Zhao, Z., Guttmann, J., and Möller, K., 2012, “Adaptive SLICE Method: An Enhanced Method to Determine Nonlinear Dynamic Respiratory System Mechanics,” Physiol. Meas., 33(1), pp. 51–64. 10.1088/0967-3334/33/1/51 22155927

[159] Mayer, H., Zaenker, K. S., and An der Heiden, U., 1995, “A Basic Mathematical Model of the Immune Response,” Chaos, 5(1), pp. 155–161. 10.1063/1.166098 12780168

[160] Stark, J., Chan, C., and George, A. J. T., 2007, “Oscillations in the Immune System,” Immunol. Rev., 216(1), pp. 213–231. 10.1111/j.1600-065X.2007.00501.x 17367345

[161] Nowak, M. A., and May, R. M., 2000, Virus Dynamics: Mathematical Principles of Immunology and Virology, Oxford University Press, Oxford, UK.

[162] Perelson, A. S., 2002, “Modelling Viral and Immune System Dynamics,” Nat. Rev. Immunol., 2(1), pp. 28–36. 10.1038/nri700 11905835

[163] Alharbi, S. A., and Rambely, A. S., 2020, “Dynamic Behaviour and Stabilisation to Boost the Immune System by Complex Interaction Between Tumour Cells and Vitamins Intervention,” Adv. Diff. Eqs., 2020(1), p. 412. 10.1186/s13662-020-02869-6

[164] Ruan, S., 2021, “Nonlinear Dynamics in Tumor-Immune System Interaction Models With Delays,” Discrete Contin. Dyn. Syst. B, 26(1), pp. 541–602. 10.3934/dcdsb.2020282

[165] Kuznetsov, V. A., Makalkin, I. A., Taylor, M. A., and Perelson, A. S., 1994, “Nonlinear Dynamics of Immunogenic Tumors: Parameter Estimation and Global Bifurcation Analysis,” Bull. Math. Biol., 56(2), pp. 295–321. 10.1007/BF02460644 8186756

[166] Kirschner, D., and Panetta, J. C., 1998, “Modeling Immunotherapy of the Tumor–Immune Interaction,” J. Math. Biol., 37(3), pp. 235–252. 10.1007/s002850050127 9785481

[167] Kiecolt Glaser, J. K., McGuire, L., Robles, T. F., and Glaser, R., 2002, “Psychoneuroimmunology: Psychological Influences on Immune Function and Health,” J. Consul. Clin. Psychol., 70(3), p. 537. 10.1037/0022-006X.70.3.537 12090368

[168] Gonzalez Herrero, M. E., and Kuehn, C., 2021, “A Qualitative Mathematical Model of the Immune Response Under the Effect of Stress,” Chaos, 31(6), p. 061104. 10.1063/5.0055784 34241308

[169] Ma, Z., Luo, Y., Zeng, C., and Zheng, B., 2022, “Spatiotemporal Diffusion as Early Warning Signal for Critical Transitions in Spatial Tumor-Immune System With Stochasticity,” PRRESEARCH, 4(2), p. 023039. 10.1103/PhysRevResearch.4.023039

[170] López, A. G., Seoane, J. M., and Sanjuán, M. A. F., 2017, “Dynamics of the Cell-Mediated Immune Response to Tumour Growth,” Philos. Trans. R. Soc. A Math. Phys. Eng. Sci., 375(2096), p. 20160291. 10.1098/rsta.2016.0291 PMC5434081 28507236

[171] Sontag, E. D., 2017, “A Dynamic Model of Immune Responses to Antigen Presentation Predicts Different Regions of Tumor or Pathogen Elimination,” Cell Syst., 4(2), pp. 231–241. 10.1016/j.cels.2016.12.003 28131824 PMC5323365

[172] de Boer, R. J., and Perelson, A. S., 1995, “Towards a General Function Describing T Cell Proliferation,” J. Theor. Biol., 175(4), pp. 567–576. 10.1006/jtbi.1995.0165 7475092

[173] de Boer, R. J., and Perelson, A. S., 2005, “Estimating Division and Death Rates From CFSE Data,” J. Comput. Appl. Math., 184(1), pp. 140–164. 10.1016/j.cam.2004.08.020 16832737

[174] Hodgkin, A. L., and Huxley, A. F., 1952, “A Quantitative Description of Membrane Current and Its Application to Conduction and Excitation in Nerve,” J. Physiol., 117(4), pp. 500–544. 10.1113/jphysiol.1952.sp004764 12991237 PMC1392413

[175] Wilson, H. R., and Cowan, J. D., 1972, “Excitatory and Inhibitory Interactions in Localized Populations of Model Neurons,” Biophys. J., 12(1), pp. 1–24. 10.1016/S0006-3495(72)86068-5 4332108 PMC1484078

[176] Freeman, W. J., 2000, Neurodynamics: An Exploration in Mesoscopic Brain Dynamics, Springer, New York.

[177] Breakspear, M., 2017, “Dynamic Models of Large-Scale Brain Activity,” Nat. Neurosci., 20(3), pp. 340–352. 10.1038/nn.4497 28230845

[178] Lytton, W. W., Arle, J., Bobashev, G., Ji, S., Klassen, T. L., Marmarelis, V. Z., Schwaber, J., Sherif, M. A., and Sanger, T. D., 2017, “Multiscale Modeling in the Clinic: Diseases of the Brain and Nervous System,” Brain Inf., 4(4), pp. 219–230. 10.1007/s40708-017-0067-5 PMC5709279 28488252

[179] Stam, C. J., Jones, B. F., Manshanden, I., van Cappellen van Walsum, A. M., Montez, T., Verbunt, J. P. A., de Munck, J. C., van Dijk, B. W., Berendse, H. W., and Scheltens, P., 2006, “Magnetoencephalographic Evaluation of Resting-State Functional Connectivity in Alzheimer's Disease”, Neuroimage, 32(3), pp. 1335–1344. 10.1016/j.neuroimage.2006.05.033 16815039

[180] Moshé, S. L., Perucca, E., Ryvlin, P., and Tomson, T., 2015, “Epilepsy: New Advances,” Lancet, 385(9971), pp. 884–898. 10.1016/S0140-6736(14)60456-6 25260236

[181] Pijn, J. P., Velis, D. N., van der Heyden, M. J., DeGoede, J., van Veelen, C. W., and Lopes da Silva, F. H., 1997, “Nonlinear Dynamics of Epileptic Seizures on Basis of Intracranial EEG Recordings,” Brain Topograp., 9(4), pp. 249–270. 10.1007/BF01464480 9217984

[182] Hagemann, A., Wilting, J., Samimizad, B., Mormann, F., and Priesemann, V., 2021, “Assessing Criticality in Pre-Seizure Single-Neuron Activity of Human Epileptic Cortex,” PLoS Comput. Biol., 17(3), p. e1008773. 10.1371/journal.pcbi.1008773 33684101 PMC7971851

[183] Granger, C. W. J., 1969, “Investigating Causal Relations by Econometric Models and Cross-Spectral Methods,” Econometrica, 37(3), pp. 424–438. 10.2307/1912791

[184] Seth, A. K., Barrett, A. B., and Barnett, L., 2015, “Granger Causality Analysis in Neuroscience and Neuroimaging,” J. Neurosci., 35(8), pp. 3293–3297. 10.1523/JNEUROSCI.4399-14.2015 25716830 PMC4339347

[185] Burioka, N., Miyata, M., Cornélissen, G., Halberg, F., Takeshima, T., Kaplan, D. T., Suyama, H., ., 2005, “Approximate Entropy in the Electroencephalogram During Wake and Sleep,” Clin. EEG Neurosci., 36(1), pp. 21–24. 10.1177/155005940503600106 15683194 PMC2563806

[186] Jeong, J., 2004, “EEG Dynamics in Patients With Alzheimer's Disease,” Clin. Neurophysiol., 115(7), pp. 1490–1505. 10.1016/j.clinph.2004.01.001 15203050

[187] Mizuno, T., Takahashi, T., Cho, R. Y., Kikuchi, M., Murata, T., Takahashi, K., and Wada, Y., 2010, “Assessment of EEG Dynamical Complexity in Alzheimer's Disease Using Multiscale Entropy,” Clin. Neurophysiol., 121(9), pp. 1438–1446. 10.1016/j.clinph.2010.03.025 20400371 PMC2914820

[188] Maestú, F., Babiloni, C., Başar, E., ., 2015, “Brain Oscillations in Alzheimer's Disease: EEG Markers for Diagnosis and Progression,” Int. J. Alzheimer's Disease, 2015, p. 172419.

[189] Stam, C. J., and van Dijk, B. W., 2002, “Synchronization Likelihood: An Unbiased Measure of Generalized Synchronization in Multivariate Data Sets,” Phys. D Nonlinear Phenom., 163(3–4), pp. 236–251. 10.1016/S0167-2789(01)00386-4

[190] Stoffers, D., Bosboom, J. L. W., Deijen, J. B., Wolters, E. C., Berendse, H. W., and Stam, C. J., 2007, “Slowing of Oscillatory Brain Activity is a Stable Characteristic of Parkinson's Disease Without Dementia,” Brain, 130(7), pp. 1847–1860. 10.1093/brain/awm034 17412733

[191] Bellman, R. E., 1961, Adaptive Control Processes: A Guided Tour, Princeton University Press, Princeton, NJ.

[192] Bishop, C. M., 2006, Pattern Recognition and Machine Learning, Springer, New York.

[193] LeCun, Y., Bengio, Y., and Hinton, G., 2015, “Deep Learning,” Nature, 521(7553), pp. 436–444. 10.1038/nature14539 26017442

[194] Esteva, A., Robicquet, A., Ramsundar, B., Kuleshov, V., DePristo, M., Chou, K., Cui, C., Corrado, G., Thrun, S., and Dean, J., 2019, “A Guide to Deep Learning in Healthcare,” Nat. Med., 25(1), pp. 24–29. 10.1038/s41591-018-0316-z 30617335

[195] Yang, Z., Huang, Y., Jiang, Y., Sun, Y., Zhang, Y.-J., and Luo, P., 2018, “Clinical Assistant Diagnosis for Electronic Medical Record Based on Convolutional Neural Network,” Sci. Rep., 8(1), p. 6329. 10.1038/s41598-018-24389-w 29679019 PMC5910396

[196] Goldberger, A. L., Amaral, L. A. N., Glass, L., Hausdorff, J. M., Ivanov, P. C., Mark, R. G., Mietus, J. E., Moody, G. B., Peng, C.-K., and Stanley, H. E., 2000, “PhysioBank, PhysioToolkit, and PhysioNet,” Circulation, 101(23), pp. e215–e220. 10.1161/01.CIR.101.23.e215 10851218

[197] Johnson, A. E. W., Pollard, T. J., Shen, L., Lehman, L.-W. H., Feng, M., Ghassemi, M., Moody, B., Szolovits, P., Celi, L. A., and Mark, R. G., 2016, “MIMIC-III, a Freely Accessible Critical Care Database,” Sci. Data, 3(1), p. 160035. 10.1038/sdata.2016.35 27219127 PMC4878278

[198] Cross, Z. R., Gutman, D. A., and Madabhushi, A., 2022, “Decoding Tissue Architecture With Deep Learning and Nonlinear Feature Analysis,” Nat. Biomed. Eng., 6(7), pp. 742–755.

[199] Zhang, Y., Li, X., Cheng, J., and Xu, M., 2023, “Fractal and Entropy-Based Deep Learning for Medical Image Characterization: A Review,” Med. Image Anal., 88, p. 102880.37413792

[200] Karniadakis, G. E., Kevrekidis, I. G., Lu, L., Perdikaris, P., Wang, S., and Yang, L., 2021, “Physics-Informed Machine Learning,” Nat. Rev. Phys., 3(6), pp. 422–440. 10.1038/s42254-021-00314-5

[201] Raissi, M., Perdikaris, P., and Karniadakis, G. E., 2019, “Physics-Informed Neural Networks: A Deep Learning Framework for Solving Forward and Inverse Problems Involving Nonlinear Partial Differential Equations,” J. Comput. Phys., 378, pp. 686–707. 10.1016/j.jcp.2018.10.045

[202] Zhou, B., Liu, Y., Wang, Z., Jin, Q., and Zhang, J., 2021, “Self-Supervised Learning for ECG Representation Via Temporal and Morphological Contrastive Learning,” 29th ACM International Conference on Multimedia (ACM MM), Chengdu, China, Oct. 20–24, pp. 1452–1461.

[203] Saeed, A., Grinsztajn, S., Johnson, A. E. W., Badawi, O., Delétang, G., Celi, L. A., Ré, C., ., 2023, “Self-Supervised Representation Learning for Multimodal Health Data,” Nat. Biomed. Eng., 7(10), pp. 1221–1235.

[204] Mohamad, T. H., Abbasi, A., Kappaganthu, K., and Nataraj, C., 2023, “On Extraction, Ranking and Selection of Data-Driven and Physics-Informed Features for Bearing Fault Diagnostics,” Knowl.-Based Syst., 276, p. 110744. 10.1016/j.knosys.2023.110744

[205] Bai, S., Kolter, J. Z., and Koltun, V., 2018, “An Empirical Evaluation of Generic Convolutional and Recurrent Networks for Sequence Modeling,” 35th International Conference on Machine Learning (ICML), Stockholm, Sweden, July 10--15, pp. 473–482.

[206] Vaswani, A., Shazeer, N., Parmar, N., Uszkoreit, J., Jones, L., Gomez, A. N., Kaiser, Ł., and Polosukhin, I., 2017, “Attention is All You Need,” 31st Conference on Neural Information Processing Systems (NIPS), Long Beach, CA, Dec. 4–9, pp. 5998--6008.https://arxiv.org/pdf/1706.03762

[207] Chen, R. T. Q., Rubanova, Y., Bettencourt, J., and Duvenaud, D., 2018, “Neural Ordinary Differential Equations,” Adv. Neural Inf. Process. Syst. (NeurIPS), Montréal, QC, Canada, Dec. 3–8, pp. 6571–6583. 10.48550/arXiv.1806.07366

[208] Gal, Y., and Ghahramani, Z., 2016, “Dropout as a Bayesian Approximation: Representing Model Uncertainty in Deep Learning,” 33rd International Conference on Machine Learning (ICML), New York, June 19–24, pp. 1050–1059. 10.48550/arXiv.1506.02142

[209] Vovk, V., Gammerman, A., and Shafer, G., 2022, Algorithmic Learning in a Random World: Conformal Prediction Framework, Springer, Cham, Switzerland.

[210] Ghorbanian, P., Ghaffari, A., Jalali, A., and Nataraj, C., 2012, “An Improved Procedure for Detection of Heart Arrhythmias With Novel Preprocessing Techniques,” Expert Syst., 29(5), pp. 478–491. 10.1111/j.1468-0394.2011.00606.x

[211] Jalali, A., Nataraj, C., Butchy, M., and Ghaffari, A., 2011, “Feature Extraction and Abnormality Detection in Autonomic Regulation of Cardiovascular System,” ASME Paper No. DETC2011-48617. 10.1115/DETC2011-48617

[212] Jalali, A., Buckley, E. M., Lynch, J. M., Schwab, P. J., Licht, D. J., and Nataraj, C., 2014, “Prediction of Periventricular Leukomalacia Occurrence in Neonates After Heart Surgery,” IEEE J. Biomed. Health Inf., 18(4), pp. 1453–1460. 10.1109/JBHI.2013.2285011 PMC4122287 24122606

[213] Jalali, A., Berg, R., Nadkarni, V., and Nataraj, C., 2012, “Model Based Optimization of The Cardiopulmonary Resuscitation (CPR) Procedure,” 34th International Conference of the IEEE Engineering in Medicine and Biology in Medicine, San Diego, California, Aug. 28–Sept. 1, pp. 4986–4989. 10.1109/EMBC.2012.6346031 23365992

[214] Hannun, A. Y., Rajpurkar, P., Haghpanahi, M., Tison, G. H., Bourn, C., Turakhia, M. P., and Ng, A. Y., 2019, “Cardiologist-Level Arrhythmia Detection and Classification in Ambulatory Electrocardiograms Using a Deep Neural Network,” Nat. Med., 25(1), pp. 65–69. 10.1038/s41591-018-0268-3 30617320 PMC6784839

[215] Attia, Z. I., Noseworthy, P. A., Lopez-Jimenez, F., Asirvatham, S. J., Deshmukh, A. J., Gersh, B. J., Carter, R. E., ., 2019, “An Artificial Intelligence–Enabled ECG Algorithm for the Identification of Patients With Atrial Fibrillation During Sinus Rhythm: A Retrospective Analysis of Outcome Prediction,” Lancet, 394(10201), pp. 861–867. 10.1016/S0140-6736(19)31721-0 31378392

[216] Bender, D., Morgan, R. W., Nadkarni, V. M., Berg, R. A., Zhang, B., Kilbaugh, T. J., Sutton, R. M., and Nataraj, C., 2021, “MLWAVE: A Novel Algorithm to Classify Primary Versus Secondary Asphyxia-Associated Ventricular Fibrillation,” Resuscit. Plus, 5, p. 100052. 10.1016/j.resplu.2020.100052 PMC7869586 33569548

[217] Bender, D., Nadkarni, V. M., and Nataraj, C., 2020, “A Machine Learning Algorithm to Improve Patient-Centric Pediatric Cardiopulmonary Resuscitation,” Inf. Med. Unlocked, 19, p. 100339. 10.1016/j.imu.2020.100339

[218] Rudin, C., Chen, C., Chen, Z., Huang, H., Semenova, L., and Zhong, C., 2022, “Interpretable Machine Learning: Fundamental Principles and 10 Grand Challenges,” Stat. Surv., 16, pp. 1–85. 10.48550/arXiv.2103.11251

[219] Nataraj, C., 2025, “How Can Nonlinear Dynamics Complement Machine Learning?,” Nonlinear Dyn., (accepted). 10.52843/cassyni.ldfhrf

[220] Liu, Z., Kambali, P. N., and Nataraj, C., 2025, “Hybrid Adaptive Modeling Using Neural Networks Trained With Nonlinear Dynamics Based Features,” Knowl.-Based Syst., 323, p. 113674. 10.1016/j.knosys.2025.113674

[221] Glaessgen, E. H., and Stargel, D. S., 2012, “The Digital Twin Paradigm for Future NASA and U.S. Air Force Vehicles,” AIAA Paper No. 6.2012-1818. 10.2514/6.2012-1818

[222] Tao, F., Zhang, M., Liu, A., and Nee, A. Y. C., 2018, “Digital Twin Driven Smart Manufacturing: Connotation, Reference Model, Applications and Research Issues,” CIRP Ann., 67(2), pp. 647–669.

[223] National Academies of Sciences, Engineering and Medicine, 2024, Foundational Research Gaps and Future Directions for Digital Twins, The National Academies Press, Washington, DC. 39088664

[224] Coveney, P. V., Diaz, V., Hunter, P., Kohl, P., and Viceconti, M., 2011, “The Virtual Physiological Human,” Interface Focus, 1(3), pp. 281–285. 10.1098/rsfs.2011.0020

[225] Hoekstra, A. G., van Bavel, E., Siebes, M., Gijsen, F., and Geris, L., 2018, “Virtual Physiological Human 2016: Translating the Virtual Physiological Human to the Clinic,” Interface Focus, 8(1), p. 20170067. 10.1098/rsfs.2017.0067

[226] Fullwood, D. T., Jensen, B. D., Weaver, B., Kölling, S., Sparks, A., and Regez, B., 2019, “Digital Twins in Healthcare: The Implications of Dynamic, Patient-Specific Modeling,” Appl. Sci., 9(23), p. 4824. 10.3390/app9234824

[227] Corral-Acero, J., Margara, F., Marciniak, M., Rodero, C., Loncaric, F., Feng, Y., Gilbert, A., ., 2020, “The Digital Twin to Enable the Vision of Precision Cardiology,” Eur. Heart J. Dig. Health, 1(1), pp. 4–19. 10.1093/eurheartj/ehaa159 PMC7774470 32128588

[228] Sel, K., Osman, D., Zare, F., Masoumi Shahrbabak, S., Brattain, L., Hahn, J.-O., Inan, O. T., ., 2024, “Building Digital Twins for Cardiovascular Health: From Principles to Clinical Impact,” J. Am. Heart Assoc., 13(19), p. e031981. 10.1161/JAHA.123.031981 39087582 PMC11681439

[229] Chabiniok, R., Wang, V. Y., Hadjicharalambous, M., Asner, L., Lee, J., Sermesant, M., Kuhl, E., ., 2016, “Multiphysics and Multiscale Modelling, Data–Model Fusion and Integration of Organ Physiology in the Clinic: Ventricular Cardiac Mechanics,” Interface Focus, 6(2), p. 20150083. 10.1098/rsfs.2015.0083 27051509 PMC4759748

[230] Björnsson, B., Borrebaeck, C., Elander, N., Gasslander, T., Gawel, D. R., Gustafsson, M., Jörnsten, R., Nystedt, S., Tegnér, J., and Nilsson, P., 2023, “Digital Twins to Personalize Medicine,” Genome Med., 15, p. 18. 10.1186/s13073-019-0701-3 31892363 PMC6938608

[231] Rudnicka, Z., Rudnicki, R., Kolasa, K., Tyburek, K., Smolen, A., and Ozimek, P., 2024, “Cardiac Healthcare Digital Twins Supported by Artificial Intelligence: A Review (2020–2024),” Electronics, 13(5), p. 866. 10.3390/electronics13050866

[232] Nickerson, D., Atalag, K., de Bono, B., Geiger, J., Goble, C., Hollmann, S., Lonien, J., ., 2016, “The Human Physiome: How Standards, Software and Innovative Service Infrastructures Are Providing the Building Blocks to Make It Achievable,” Interface Focus, 6(2), p. 20150103. 10.1098/rsfs.2015.0103 27051515 PMC4759754

[233] Pathmanathan, P., and Gray, R. A., 2019, “Validation and Verification of Computational Models of Cardiac Physiology,” Front. Physiol., 10, p. 127.30828305

[234] Gray, R. A., and Pathmanathan, P., 2016, “Patient-Specific Cardiovascular Computational Modeling: Diversity of Personalization and Challenges,” J. Cardiovasc. Transl. Res., 9, pp. 197–206. 10.1007/s12265-018-9792-2 PMC5908828 29512059

[235] Masison, J., Sun, S., Newton, A., Gray, R. A., Yu, T., and Edelstein-Keshet, L., 2021, “Hub-and-Spoke Architectures for Integrating Multiscale Models of Human Physiology,” Front. Physiol., 12, p. 661695.

[236] Ching, T., Himmelstein, D. S., Beaulieu-Jones, B. K., Kalinin, A. A., Do, B. T., Way, G. P., Ferrero, E., ., 2018, “Opportunities and Obstacles for Deep Learning in Biology and Medicine”, J. R. Soc. Interface., 15(141), p. 20170387. 10.1098/rsif.2017.0387 29618526 PMC5938574

[237] Topol, E. J., 2019, “High-Performance Medicine: The Convergence of Human and Artificial Intelligence,” Nat. Med., 25(1), pp. 44–56. 10.1038/s41591-018-0300-7 30617339

[238] Beam, A. L., and Kohane, I. S., 2018, “Big Data and Machine Learning in Health Care,” JAMA, 319(13), pp. 1317–1318. 10.1001/jama.2017.18391 29532063

[239] Rajkomar, A., Dean, J., and Kohane, I., 2019, “Machine Learning in Medicine,” New Engl. J. Med., 380(14), pp. 1347–1358. 10.1056/NEJMra1814259 30943338

[240] Miotto, R., Wang, F., Wang, S., Jiang, X., and Dudley, J. T., 2018, “Deep Learning for Healthcare: Review, Opportunities and Challenges,” Brief. Bioinform., 19(6), pp. 1236–1246. 10.1093/bib/bbx044 28481991 PMC6455466

[241] Theilman, B. H., Aimone, J. B., 2025, “Solving Sparse Finite Element Problems on Neuromorphic Hardware”, Nat. Mach. Intell. (accepted) 10.1038/s42256-025-01143-2

[242] Xue, Y., Zhang, X., Huang, X., Chen, H., Qi, X., Feng, Y., and Zhuang, X., 2023, “Generative Diffusion Models for Medical Image Augmentation: Opportunities and Challenges,” IEEE Trans. Med. Imag. (accepted).

[243] Armanious, K., Jiang, C., Fischer, M., Küstner, T., Hepp, T., Nikolaou, K., Gatidis, S., and Yang, B., 2020, “MedGAN: Medical Image Translation Using GANs,” Comput. Med. Imag. Graph., 79, p. 101684. 10.1016/j.compmedimag.2019.101684 31812132

[244] van Herten, R. L. M., Scannell, C. M., Plein, S., Ferreira, V. M., Ismail, T. F., and Hamrud, E., 2022, “Physics-Informed Neural Networks for Myocardial Perfusion Magnetic Resonance Quantification,” Med. Image Anal., 81, p. 102545. 10.1016/j.media.2022.102399 PMC9051528 35299005

[245] Sel, K., Mohammadi, A., Pettigrew, R. I., and Jafari, R., 2023, “Physics-Informed Neural Networks for Modeling Physiological Time Series for Cuffless Blood Pressure Estimation,” NPJ Dig. Med., 6(1), p. 102. 10.1038/s41746-023-00853-4 PMC10256762 37296218

[246] Zhao, A., Zhang, Y., Chen, X., Li, P., and Wang, H., 2025, “Physics-Informed Neural Networks for Physiological Signal Analysis: Principles, Applications, and Challenges,” Physiol. Meas. (In Press). 10.1088/1361-6579/adf1d3 PMC12308510 40680769

[247] Chen, T., Kornblith, S., Norouzi, M., and Hinton, G., 2020, “A Simple Framework for Contrastive Learning of Visual Representations,” International Conference on Machine Learning (ICML), Virtual Conference, July 13–18, pp. 1597–1607.

[248] Bardes, A., Ponce, J., and LeCun, Y., 2022, “VICReg: Variance-Invariance-Covariance Regularization for Self-Supervised Learning,” International Conference on Learning Representations (ICLR), Virtual Conference, Apr. 25–29. 10.48550/arXiv.2105.04906

[249] Assran, M., LeJeune, D., Misra, I., Jurafsky, D., LeCun, Y., and Ma, T., 2023, “Self-Supervised Learning From Time Series With Asymmetric Contrastive Loss,” Advances in Neural Information Processing Systems (NeurIPS), New Orleans, LA, Dec. 10–16.

[250] Chen, R. T. Q., Rubanova, Y., Bettencourt, J., and Duvenaud, D., 2018, “Neural Ordinary Differential Equations,” Advances in Neural Information Processing Systems 31 (NeurIPS), Montréal, QC, Canada, Dec. 3–8, pp. 6571–6583.https://papers.nips.cc/paper/2018/hash/69386f6bb1dfed68692a24c8686939b9-Abstract.html

[251] Li, X., Wong, T., Chen, R., and Duvenaud, D., 2020, “Scalable Gradients and Adaptive Solvers for Stochastic Differential Equations,” , Advances in Neural Information Processing Systems (NeurIPS), Virtual Conference, Dec. 6–12, pp. 11762–11774.

[252] Daryakenari, N. A., Bagheri, M., Taghvaei, A., and Karniadakis, G. E., 2024, “AI-Aristotle: A Physics-Informed Framework for Systems Biology Gray-Box Identification,” PLoS Comput. Biol., 20(3), p. e1011916. 10.1371/journal.pcbi.1011916 38470870 PMC10931529

[253] Yazdani, A., Lu, L., Raissi, M., and Karniadakis, G. E., 2020, “Systems Biology Informed Deep Learning for Inferring Dynamics of Biological Systems,” PLoS Comput. Biol., 16(11), p. e1007575. 10.1371/journal.pcbi.1007575 33206658 PMC7710119

[254] Chen, Z., Liu, Y., and Sun, H., 2021, “Physics-Informed Learning of Governing Equations From Scarce Data,” Nat. Commun., 12(1), p. 6136. 10.1038/s41467-021-26434-1 34675223 PMC8531004

[255] Abbasi, A., Kambali, P. N., Shahidi, P., and Nataraj, C., 2024, “Physics-Informed Machine Learning for Modeling Multidimensional Dynamics of Coupled Nonlinear Systems,” Nonlinear Dyn., 112(24), pp. 21565–21585. 10.1007/s11071-024-10163-3

[256] Islam, N. N., Coban, M. A., Fuller, J. M., Weber, C., Chitale, R., Jussila, B., Brock, T. J., Tao, C., and Caulfield, T. R., 2025, “Dynamicasome—A Molecular Dynamics-Guided and AI-Driven Pathogenicity Prediction Catalogue for All Genetic Mutations,” Commun. Biol., 8(1), p. 958. 10.1038/s42003-025-08334-y 40624350 PMC12234709

